# Classification of porcine reproductive and respiratory syndrome clinical impact in Ontario sow herds using machine learning approaches

**DOI:** 10.3389/fvets.2023.1175569

**Published:** 2023-06-07

**Authors:** Akshay Chadha, Rozita Dara, David L. Pearl, Daniel Gillis, Thomas Rosendal, Zvonimir Poljak

**Affiliations:** ^1^School of Computer Science, University of Guelph, Guelph, ON, Canada; ^2^Department of Population Medicine, Ontario Veterinary College, University of Guelph, Guelph, ON, Canada; ^3^SVA, National Veterinary Institute, Uppsala, Sweden

**Keywords:** PRRS, pathogenicity, classifcation, machine learning, ORF5 gene

## Abstract

Since the early 1990s, porcine reproductive and respiratory syndrome (PRRS) virus outbreaks have been reported across various parts of North America, Europe, and Asia. The incursion of PRRS virus (PRRSV) in swine herds could result in various clinical manifestations, resulting in a substantial impact on the incidence of respiratory morbidity, reproductive loss, and mortality. Veterinary experts, among others, regularly analyze the PRRSV open reading frame-5 (ORF-5) for prognostic purposes to assess the risk of severe clinical outcomes. In this study, we explored if predictive modeling techniques could be used to identify the severity of typical clinical signs observed during PRRS outbreaks in sow herds. Our study aimed to evaluate four baseline machine learning (ML) algorithms: logistic regression (LR) with ridge and lasso regularization techniques, random forest (RF), k-nearest neighbor (KNN), and support vector machine (SVM), for the clinical impact classification of ORF-5 sequences and demographic data into high impact and low impact categories. First, baseline classifiers were evaluated using different input representations of ORF-5 nucleotides, amino acid sequences, and demographic data using a 10-fold cross-validation technique. Then, we designed a consensus voting ensemble approach to aggregate the different types of input representations for genetic and demographic data for classifying clinical impact. In this study, we observed that: (a) for abortion and pre-weaning mortality (PWM), different classifiers gained improvement over baseline accuracy, which showed the plausible presence of both genotypic-phenotypic and demographic-phenotypic relationships, (b) for sow mortality (SM), no baseline classifier successfully established such linkages using either genetic or demographic input data, (c) baseline classifiers showed good performance with a moderate variance of the performance metrics, due to high-class overlap and the small dataset size used for training, and (d) the use of consensus voting ensemble techniques helped to make the predictions more robust and stabilized the performance evaluation metrics, but overall accuracy did not substantially improve the diagnostic metrics over baseline classifiers.

## 1. Introduction

Porcine reproductive and respiratory syndrome (PRRS) is currently considered the most important endemic viral disease affecting commercial swine populations in North America (1, 2). PRRS is present globally, with only a few countries with swine populations in Europe (i.e., Switzerland, Sweden, Norway, and Finland) and New Zealand free from it (3, 4). PRRS affects the pork industry’s breeding and grower-finisher sectors through a significant decrease in reproductive health, an increase in mortality and morbidity, and a reduction in growth rate (5). In individual animals, the disease is characterized by reproductive and respiratory clinical signs such as abortion, anorexia, dyspnea, inappetence, premature farrowing, stillborn or weak-born piglets, mummified fetuses, and increased likelihood of mortality (5–7). The causative agent of PRRS is the porcine reproductive and respiratory syndrome virus (PRRSV), an RNA virus classified into the family Arteriviridae, now classified into the genus Rodartevirus (8). PRRSV is classified into two major types: the European type (Type-1 or Lelystad virus) and the North American type (Type-2 or VR-2332) (9–11).

PRRSV diagnostics and monitoring depend on molecular characterization, commonly based on the 603 nucleotide base pairs long open reading frame 5 (ORF-5) region, which encodes for envelope surface glycoprotein (GP5). The sequencing of ORF-5 is used to establish the plausible origin of outbreaks and for conducting molecular and epidemiological studies (12–14). Once available, the ORF-5 nucleotide sequence is used to classify PRRSV into distinct groups, either based on the in-silico classification of ORF-5 into discrete restricted fragment length polymorphism (RFLP) patterns or genetic clusters based on the direct application of clustering algorithms (14). Although PRRSV circulates endemically, temporal emergence and clustering of individual genotypes are well established. Some of the distinct PRRS virus groups that emerged consistently have a high clinical impact in swine herds, and this assessment is typically done using clinical impressions and in some cases, followed by experimental studies (12, 15, 16). Some recent examples of PRRS viruses with a reported high clinical impact are designated as RFLPs 1–7–4 (17), 1–8–4 (13, 18, 19), 1–22–2 (8), and RLFP 1–4–2 or 1–4–4 (20). Apart from ORF-5 genetic sequences, herd-level demographic attributes are also known to contribute to the clinical severity of outbreaks (5). Therefore, classifying PRRSV circulating strains helps predict the possible clinical impact of the outbreak in a herd or region. This prognostic assessment is essential because the management of PRRSV in sow herds is complex and involves several pharmaceuticals, demographic, and management approaches used in different combinations and sequences (21–23).

Understanding the likely clinical impact of PRRSV outbreaks using demographic and genetic data could potentially influence the timing of intervention strategies for managing PRRSV outbreaks and reducing their impact on health, welfare, and productivity. The wide availability of nucleotide data, when coupled with advances in machine learning (ML) approaches, creates unique opportunities to explore the clinical prognosis of PRRSV outbreaks using a data-driven approach. ML techniques have been used with different representations of genomic sequences for sequence classification tasks (24–31), including the pathogenicity classification of avian influenza viruses in poultry (32). An overview of ML and ensemble approaches used in this study and different bioinformatics applications can be found in Section S1 (see Supplementary materials).

This study aimed to explore the application of supervised ML techniques for predicting the clinical impact of PRRSV in sow herds. This was accomplished by addressing two objectives. The first objective was to evaluate the accuracy of different baseline ML algorithms using ORF-5 sequence data and herd-level demographic and management factors data for the prognosis of three distinct and frequently reported clinical impacts at the herd level: abortion, preweaning mortality (PWM), and sow mortality (SM). The second objective was to create an ensemble using the best-performing baseline algorithms on different input representations and compare its performance with the individual classifiers.

## 2. Materials and methods

### 2.1. Data collection, data cleaning, and data preprocessing

The ORF-5 sequence and the demographic dataset were collected during a previous study by Rosendal et al. (33), for which sequencing was performed at the Animal Health Laboratory (AHL), University of Guelph, Guelph, Ontario, Canada. Briefly, the samples for sequencing were collected from swine herds in Ontario and submitted to the AHL from Sept. 1, 2004, to Aug. 31, 2007. Dichotomous herd-level information about the presence of different clinical signs for each herd (i.e., abortion, sows-off-feed, stillborn pigs, weak born pigs, sow/boar mortality, pre-weaning mortality, nursery respiratory, nursery mortality, finisher respiratory, and finisher mortality) were collected by a retrospective telephone interview with the owner or manager of the respective herds (33). In the literature, it is noted that PRRS clinical signs vary and may include a range of respiratory signs, reproductive signs, reduced growth rate, and neurological signs (5, 6, 34). The decision to include these three clinical signs and production measures was based on several criteria. First, these clinical signs are frequently reported to accompany different phenotypes of PRRSV strains (i.e., reproductive and respiratory). Second, they are important production, health, and welfare indicators for animals and producers. Third, it was deemed that such clinical signs will have the lowest likelihood of misclassification among all clinical signs originally available from the previous study, and it would plausibly represent a reasonable number of the clinical impact that could be managed within the framework of this computational study. Therefore, among the collected data in Rosendal et al. (33), we used three clinical signs: abortion, PWM, and SM, as the outcomes of interest for this study.

As a data-cleaning technique, we excluded the herds with missing information about any of the three clinical signs used in this study, which resulted in a total of 247 (*n* = 442) herds for analysis purposes. Furthermore, the herd demographic, management, and outbreak attributes included: the number of sows in inventory, type of herd, date of outbreak, and whether modified live vaccine (MLV) along with live virus inoculation (LVI) were included in the management of the herds during the PRRS outbreak. The month extracted from the PRRS outbreak date data was used as input for ML algorithms. The details about the type and distribution of demographic variables used in this study are described in Supplementary Table S1. The starting codon for many deoxyribose nucleic acid (DNA) sequences was missing. Therefore, the translation of nucleotide sequences into amino acid sequences was performed by manually inspecting and keeping the longest translated frames using the “Geneious 2019.0.4”^1^ tool and Expasy (35). Finally, DNA and amino acid sequences were aligned using the Clustal Omega multiple sequence alignment algorithm (36, 37).

The application of ML classification techniques requires class label information for the given inputs. In Rosendal et al. (33), the information about the clinical signs for each herd was recorded through an interview process primarily over the telephone, along with mailed/faxed survey filling and herd veterinarians administering the survey to clients. The clinical outcomes information was observed in binary format at the herd level for the circulating strains after the outbreak was detected and confirmed with a study selection criterion (33). As the circulating PRRSV strains can cause severe respiratory distress, high fever, and reproductive failure in pigs, therefore the differential impact for each clinical outcome may be linked with its genotype and herd-level demographics. In each herd, for each clinical sign, the binary observation ‘0’ indicated an absence of a specific clinical sign in association with the PRRS strain outbreak, and ‘1’ represented otherwise. Therefore, for classification purposes in this study, the binary outcome labels were categorized into low-impact (LI) and high-impact (HI) categories based on the recorded binary observations. The distribution of LI and HI class labels and the frequency of the majority class for DNA sequences, amino acid sequences, and demographic dataset for each clinical sign used in this study are shown in Table 1. Table 1 shows that the LI/HI class distribution was relatively balanced for abortion and PWM but imbalanced for SM. The baseline accuracy of a dataset is often characterized as the percentage of the majority class in the data.

**Table 1:** Distribution and baseline accuracy of PRRS DNA sequences, Amino acid sequences, and demographics data for clinical signs used in this study.

Input type	Clinical sign	Label (LI/HI)	Baseline accuracy (Frequency of majority class) (%)
DNA/Protein/Demographic	Abortion	129/118	52.22
	PWM	125/122	50.60
	Sow mortality	173/74	70.04

DNA, deoxyribose nucleic acid; PWM, pre-weaning mortality; LI, low impact; HI, high impact.

ML algorithms require a numerical representation of DNA and amino acid sequences, which do not contain explicit features. We used three encoding strategies to encode the symbolic string sequences as input to ML algorithms: (1) dummy variable encoding was used to encode the categorical information into numeric values and (2) the increased dimensionality in the number of variables with low sample size makes ML algorithms prone to overfitting, which is also known as the ‘curse of dimensionality’ (38, 39). As the ORF-5 sequences are highly dimensional, we reduced the dimensionality by using principal component analysis (PCA) based embedded representation for extracting low dimensional features of different sizes (i.e., 2, 3, 5, 10, 15, 20). PCA selects the best-performing features which overcome the issues related to the ‘curse of dimensionality.’ Finally, (3) we extracted frequency count-based features of different sizes (i.e., 2, 3, 4, 5, 6) for both DNA and amino acid sequences. Frequency count-based vectors were used in the literature as input to ML algorithms for classification problems in text mining and genomics (40–42). In addition, *k*-mer count-based encoded numeric representations of genetic sequences were used as an input to ML algorithms in the literature for classification purposes. However, for larger values of *k*, the input vector becomes highly dimensional and sparsely populated.

### 2.2. Proposed methodology

In this study, we investigated the plausible relationships between ORF-5 genotype data and herd-level demographic data with three phenotypic outcomes using the general framework of ML techniques described in Supplementary Figure S1. We also used ensemble techniques to address issues with small and complex datasets. As shown in Supplementary Figure S1, collecting data relevant to the PRRS classification problem is important. Therefore first, the data were requested from the AHL, and data-cleaning techniques were applied to generate the data for experimental purposes. Data cleaning and transformation are crucial in preparation for modeling. Therefore, as a data preprocessing step, the numeric values in demographic data were standardized before feeding it as an input to ML classifiers. Furthermore, the DNA and amino acid sequences were encoded using dummy, *k*-mer, and PCA-based feature vector input representations. Choosing appropriate machine learning models and using the data to train the model is important, and during training, the model parameters optimization is used to minimize the error between the predicted and actual outcomes. The encoded representations obtained using each format were used as input to the following linear and non-linear baseline classification algorithms: logistic regression (LR) with lasso/ridge regularization, random forest (RF), k-nearest neighbor (KNN), and support vector machine (SVM). Before training, fine-tuning the hyperparameters of the model was helpful in improving performance. The optimal hyperparameter values for each ML algorithm were evaluated using the grid search approach. Finally, the performance of different classification methods used in this study was evaluated using different metrics such as accuracy, precision, recall, F1-score, and area under the curve (AUC) using a 10-fold cross-validation technique. In this study, we used F1-score as a primary evaluation metric to deal with imbalanced data.

In general, ensemble methods aggregate the predictions observed from multiple models trained using single/multiple data sources. The differences in the predictive performance of baseline classifiers indicated that introducing diversity by selecting the best-performing classifier on different input representations of data might be a more integrative approach. In addition, applying ensemble models may pose a practical solution to increase the robustness of the predictions. However, training ensembles require more computational power and memory despite being simple to train. At the same time, for classification, a voting ensemble combines the individual predictions of the two or more supervised classifiers, most employing different representations of the input datasets used.

The consensus voting ensemble approach used in this study is described in Supplementary Figure S2. Using a consensus voting-based ensemble approach, we investigated the advantage of using heterogeneous representations of input data formats and different classification techniques for each clinical sign. The best-performing baseline classifiers for each clinical sign using different input representations in each partition were collectively used for decision-making. The consensus voting ensemble approach used in this study is described below:

Input: PRRSV genetic sequences and demographic data with clinical sign information.

Output: Prediction of PRRS clinical impact.

Step 1: Identify the best-performing classifiers and respective hyperparameters for each clinical sign (i.e., abortion, PWM, and SM) for different input representations (i.e., demographic, dummy, *k*-mer, and PCA) during each partition.

Step 2: For abortion, PWM and SM train the best-performing baseline classifiers observed during each partition using the demographic, DNA, and amino acid datasets.

Step 3: For abortion, PWM and SM aggregate the predicted outcomes using consensus voting during each partition by assigning the majority outcome obtained from DNA, amino acid, and demographic datasets.

Step 4: Evaluate the performance of each clinical sign.

### 2.3. Model evaluation criteria

We used a 10-fold cross-validation technique in which the dataset was partitioned into ten partitions, from which nine were used for training, and the remaining partition was used for evaluating the model’s performance. Before the k-fold partitioning was performed, the data was randomly shuffled so that the same data from both the HI and LI categories were shown to the classifier in each partition. As we compared the performance of multiple models, therefore, for uniformity, different representations (i.e., demographic or genetic) of the same herd were used in each partition as input for the ML models used in this study. Model overfitting is a problem that arises when a model becomes more complex than needed. For this reason, it’s crucial to find an acceptable balance between model complexity and fitting accuracy. Overfitting can be avoided with the help of grid search by checking how well the model generalizes on a separate validation set. The model parameters were selected for each partition using grid search-based hyperparameter selection (43) using internal cross-validation on the data in nine partitions. After the grid search was performed in each partition, the models were trained with the optimal parameters using which the highest evaluation metrics were observed. A brief description of the parameters evaluated for each classification method is defined in Supplementary Table S2.

In this study, we evaluated the performance of different classification models using accuracy (Acc.), sensitivity (SN), specificity (SP), and F1-score defined in (44). As reported in the literature, accuracy, sensitivity, and specificity are unreliable performance metrics when the models are developed using a complex and imbalanced dataset. In such cases, other metrics from the confusion matrix, such as precision, recall, area under the curve (AUC) score, and the F1 score, can be used to evaluate the model’s performance. AUC measures the ability of a model to discriminate between positive and negative classes. F1-score is a weighted average of the model’s precision and recall. In general, the F1-score is a useful metric when the objective is to achieve a balance between precision and recall, whereas the AUC is beneficial when the objective is to maximize the model’s ability to correctly classify positive and negative instances. As a primary evaluation metric, in this study, we used the F1 score to evaluate the overall contribution of precision and recall for selecting the best-performing classifiers. The mean and standard deviation among the observed evaluation metrics from all folds were also observed.

## 3. Results

The results obtained for the experiments in this study were executed on a Microsoft Windows-based machine with a quad-core i7 processor with a base frequency of 2.80 GHz and 16GB RAM. Python version 3.8, Numpy version 1.21.6, Scikit-learn library version 1.0.2, Scikit-learn pandas version 1.8.0, and Matplotlib visualization library version 3.2.2 (45, 46) were used for the classification and visualization purposes. The baseline classifiers were evaluated using the different parameters listed in Supplementary Table S2.

### 3.1. Performance evaluation of baseline classifiers on genotypic and demographic input representations for each clinical sign

A summary of the results observed using the best-performing baseline classifiers, their mean value of performance evaluation metrics observed over all the ten folds, and the standard deviation in metrics for each clinical sign used in this study are reported in Tables 2, 3, respectively.

**Table 2:** Best performing baseline classifiers for each clinical sign.

Data representation		Abortion	PWM	Sow mortality
Demographic		**KNN**	**RF**	**KNN**
DNA	Dummy	SVM	RF	KNN
	PCA	SVM (PC=10)	SVM (PC=3)	KNN (PC=2)
	K-mer	**RF (Kmer=5)**	**RF (Kmer=6)**	**KNN (Kmer=6)**
Protein	Dummy	SVM	SVM	KNN
	PCA	**SVM (PC=10)**	SVM (PC=5)	KNN (PC=10)
	K-mer	SVM (Kmer=3)	**LR (Kmer=5)**	**RF (Kmer=6)**

a The best-performing classifier among LR/RF/KNN/SVM for demographic, different input representations of DNA, and amino acid sequences for each clinical sign used in this study are highlighted in bold in the above table.

b DNA, deoxyribose nucleic acid; PCA, principal component analysis; LR, logistic regression; RF, random forest; KNN, k nearest neighbor; SVM, support vector machine; PWM, pre-weaning mortality; PC, principal component.

**Table 3:** Performance evaluation metrics of baseline classifiers for each clinical sign.

Input representations				Abortion (Baseline=52.22)				PWM (Baseline=50.60)							Sow mortality (Baseline=70.04)				
		Acc.	SN	SP	F1-score	AUC	Improvement	Acc.	SN	SP	F1-score	AUC	Improvement	Acc.	SN	SP	F1-score	AUC	Improvement
Demographic		**60.65 (14.81)**	**49.21 (18.84)**	**72.39 (18.68)**	**59.40 (15.18)**	**59.35 (17.18)**	**8.43**	**57.03 (13.47)**	**62.18 (20.63)**	**55.57 (13.51)**	**56.87 (13.38)**	**61.58 (12.10)**	**6.43**	**68.85 (12.09)**	**16.58 (11.92)**	**91.97 (9.62)**	**51.97 (11.50)**	**52.74 (19.25)**	−1.19
DNA	Dummy	68.33 (9.76)	80.71 (12.68)	57.36 (11.07)	67.61 (9.77)	70.63 (10.62)	16.11	61.97 (7.94)	66.83 (12.22)	58.76 (12.36)	61.37 (7.60)	68.95 (6.57)	11.37	69.33 (10.35)	10.58 (20.71)	96.06 (4.52)	46.62 (11.8)	60.26 (10.29)	−0.71
	PCA	68.33 (9.20)	80.83 (13.36)	57.43 (10.37)	67.60 (9.28)	69.32 (10.73)	16.11	61.08 (8.58)	77.30 (12.30)	44.84 (10.78)	59.26 (9.07)	63.13 (10.02)	10.48	66.47 (7.96)	13.33 (20.77)	91.41 (8.24)	46.37 (9.72)	59.97 (14.46)	−3.57
	K-mer	**69.53 (11.82)**	**76.03 (13.70)**	**65.16 (15.38)**	**69.19 (11.78)**	**71.67 (11.76)**	**17.31**	**64.35 (7.37)**	**68.89 (17.07)**	**62.39 (11.30)**	**63.80 (7.33)**	**68.26 (8.31)**	**13.75**	**62.40 (8.21)**	**49.81 (25.18)**	**68.41 (11.52)**	**55.96 (10.93)**	**63.49 (11.24)**	**−7.64**
Protein	Dummy	64.67 (12.65)	72.67 (15.20)	57.94 (14.63)	64.10 (12.37)	68.25 (11.17)	12.45	63.95 (9.58)	72.47 (14.35)	55.97 (10.77)	63.01 (9.28)	68.53 (10.35)	13.35	66.43 (5.80)	9.08 (11.50)	92.08 (8.21)	45.46 (7.53)	56.72 (11.78)	−3.61
	PCA	**68.70 (10.52)**	**77.48 (14.69)**	**62.01 (13.05)**	**68.25 (10.33)**	**68.48 (9.34)**	**16.48**	62.78 (5.70)	82.57 (8.12)	43.03 (8.24)	60.52 (6.09)	63.96 (11.55)	12.18	66.45 (7.32)	11.25 (20.79)	92.55 (7.83)	44.76 (8.77)	53.46 (9.56)	−3.59
	K-mer	67.15 (5.87)	66.10 (13.70)	69.56 (11.20)	66.60 (6.01)	69.93 (7.09)	14.93	**69.25 (6.80)**	**76.07 (11.84)**	**62.12 (6.09)**	**68.33 (6.45)**	**73.18 (4.30)**	**18.65**	**71.72 (11.40)**	**22.58 (22.18)**	**94.29 (5.97)**	**55.18 (15.04)**	**67.60 (10.04)**	1.68

a The performance evaluation of the best-performing classifier (standard deviation) among LR/RF/KNN/SVM for demographic, different input representations of DNA, and amino acid sequences for each clinical sign used in this study are highlighted in bold in the above table.

b Indicates best in the category for each input representation.

c Evaluation Metric (standard deviation).

d DNA, deoxyribose nucleic acid; PCA, principal component analysis; LR, logistic regression; RF, random forest; KNN, k nearest neighbor; SVM, support vector machine; PWM, pre-weaning mortality; Acc, accuracy; SN, sensitivity, SP, specificity.

#### 3.1.1. Results using demographic input representation

The detailed performance evaluation results of different baseline classifiers on the demographic dataset are reported in Supplementary Table S3. For abortion, we observed that among all baseline classifiers, the KNN classifier achieved the highest mean F1-score value of 59.40% (15.18) and a mean accuracy of 60.65% (14.81). For PWM, we observed that among all baseline classifiers, the RF classifier achieved the highest mean F1-score value of 56.87% (13.38) and a mean accuracy of 57.03% (13.47). For SM, we observed that among all the baseline classifiers, the KNN classifier achieved the highest mean F1-score value of 51.97% (11.50) and a mean accuracy of 68.85% (12.09). When using herd demographic attributes as input data, the best-performing baseline classifiers achieved 8.43 and 6.43% improvement (Table 3) over the baseline accuracy for abortion and PWM, respectively. However, we could not observe such improvement over the baseline for SM.

#### 3.1.2. Results using dummy input representations using DNA and amino acid sequences

The performance of different classifiers on dummy variable representations of DNA and amino acid sequences is reported in Supplementary Table S4 and S5, respectively. For example, using dummy variable representation for (1) DNA sequences as input, we observed that among all the classifiers, SVM, RF, and KNN classifiers achieved the highest mean F1-score value of 67.61% (9.77), 61.37% (7.60), and 46.62% (11.80) for abortion, PWM, and SM, respectively, (Supplementary Table S4); (2) amino acid sequences as input, we observed that among all the classifiers SVM, SVM, and KNN classifiers achieved the highest mean F1-score value of 64.10% (12.37), 63.01% (9.28), and 45.46 (7.53) for abortion, PWM, and SM, respectively, (Supplementary Table S5).

For abortion using dummy variable representation as input, the best-performing classifiers achieved 16.11 and 12.45% improvement over baseline accuracy for DNA and amino acid sequences, respectively (Table 3). For PWM using dummy variable representation as input, the best-performing classifiers achieved 11.37 and 13.35% improvement over baseline accuracy for DNA and amino acid sequences, respectively (Table 3).

In summary, for abortion, the dummy variable-based representation of DNA sequences resulted in higher mean accuracy and mean F1-score over baseline accuracy with lower variance than amino acid sequences (Table 3).

#### 3.1.3. Results using principal component representation of DNA and amino acid sequences

The detailed performance of different classifiers with a principal component embedded representation of DNA sequences for abortion, PWM, and SM are reported in Supplementary Tables S6–S8, respectively. SVM, SVM, and KNN classifiers achieved the highest F1-score and improvements over the baseline accuracy for abortion, PWM, and SM using the 10-dimensional, 3-dimensional, and 2-dimensional principal component representations (Supplementary Tables S6–S8). Using 10-dimensional and 3-dimensional PCA embedding as input representation of PRRSV DNA sequences, the SVM classifier achieved 16.11, and 10.48% mean improvement over baseline accuracy, respectively, for abortion and PWM (Table 3). However, we could not observe such improvement for SM. For SM, the performance of different classifiers with a principal component embedded representation of DNA sequences did not improve much over the baseline.

The performance of different classifiers with the principal component embedded representation of amino acid sequences for abortion, PWM, and SM are reported in Supplementary Tables S9–S11, respectively. SVM, SVM, and KNN classifiers achieved the highest F1-score and improvements over the baseline accuracy for abortion, PWM, and SM using the 10-dimensional, 5-dimensional, and 10-dimensional principal component representations (Supplementary Tables S9–S11). Using PCA representation of PRRSV amino acid sequences as input representation, the SVM classifier achieved 16.48 and 12.18% improvement over baseline accuracy for abortion and PWM, respectively (Table 3). However, for SM, the performance of different classifiers with a principal component embedded representation of amino acid sequences did not improve much over the baseline. In summary, using the PCA-based representation of DNA and amino acid sequences, the amino acid representation improved over baseline accuracy with moderate variance in the observed results.

#### 3.1.4. Results using principal *k*-mer input representation of DNA and amino acid sequences

The performance of different classifiers on the *k*-mer representation of DNA sequences for abortion, PWM, and SM are reported in Supplementary Tables S12–S14, respectively. The RF classifier achieved the highest F1-score and improvements over baseline accuracy for abortion and PWM using 5-mer, and 6-mer input representations, respectively (Supplementary Tables S12 and S13). The KNN classifier achieved the highest F1-score and improvements over baseline accuracy for SM using 6-mer input representation, respectively (Supplementary Table S14). Using the *k*-mer representation of PRRSV DNA sequences as input representation, the best-performing classifiers achieved 17.31, and 13.75% improvement over baseline accuracy for abortion, and PWM, respectively (Table 3). However, such an improvement could not be observed for SM.

The performance of different classifiers on the *k*-mer representation of amino acid sequences for abortion, PWM, and SM are reported in Supplementary Tables S15–S17, respectively. Briefly, SVM, LR, and RF classifiers achieved the highest F1-score and improvements over baseline accuracy for abortion and PWM using 3-mer, 5-mer, and 6-mer input representations (Supplementary Tables S15–S17). Using the *k*-mer representation of PRRSV amino acid sequences as input representation, the best-performing classifiers achieved 14.93, 18.65, and 1.68% improvement over baseline accuracy for abortion, PWM, and SM, respectively (Table 3).

In summary, from Table 3, we observed that among the *k*-mer-based representation of DNA and amino acid sequences, the amino acid representation achieved greater improvement over baseline accuracy and lower variance in the observed results for PWM but not for abortion.

### 3.2. Performance of the consensus voting ensemble approach

No single input representation and baseline classifier performed best for all clinical signs used in this study. Also, different predictive performances and uneven distributions of misclassified sequences justified the need for considering diverse input data formats using ensemble methods. Therefore, achieving the consensus among at least two different input data representations using a majority voting approach may increase the robustness in the decision-making of predictive techniques and introduce diversity in ensemble building. The results for the consensus voting approach for abortion, PWM, and SM are presented in Table 4. Following the application of the consensus voting classifier using demographic, DNA, and amino acid sequence representations as input: **(1)** for abortion consensus voting approach achieved a mean F1-score value of 69.62% (9.64) with 69.93% (9.71) mean accuracy; **(2)** for PWM consensus voting approach achieved a mean F1-score value of 66.00% (5.90) with 66.77% (5.85) mean accuracy; and **(3)** for SM consensus voting approach achieved a mean F1-score value of 50.59% (14.24) with 67.67% (11.63) mean accuracy. In summary, the application of the consensus voting approach improved by 17.71 and 16.17% over the baseline for abortion and PWM, respectively, but such an increase could not be observed for sow mortality. In addition, the consensus voting approach achieved lower variance in the standard deviation of mean accuracy and F1-score for abortion and PWM compared to baseline classifiers reported in Table 3.

**Table 4:** Evaluation metrics observed using voting ensemble technique.

Consensus voting	Acc.	SN	SP	F1-score	Improvement
Abortion (Baseline=52.22)	69.93 (9.71)	75.99 (15.03)	66.28 (13.34)	69.62 (9.64)	17.71
PWM (Baseline=50.60)	66.77 (5.85)	75.31 (15.55)	60.44 (10.74)	66.00 (5.90)	16.17
Sow Mortality (Baseline=70.04)	67.67 (11.63)	18.67 (21.89)	90.23 (6.77)	50.59 (14.24)	−2.37

Acc, accuracy; SN, sensitivity; SP, specificity; PWM, pre-weaning mortality.

### 3.3. Visualization of genetic sequences

**Figure 1:**
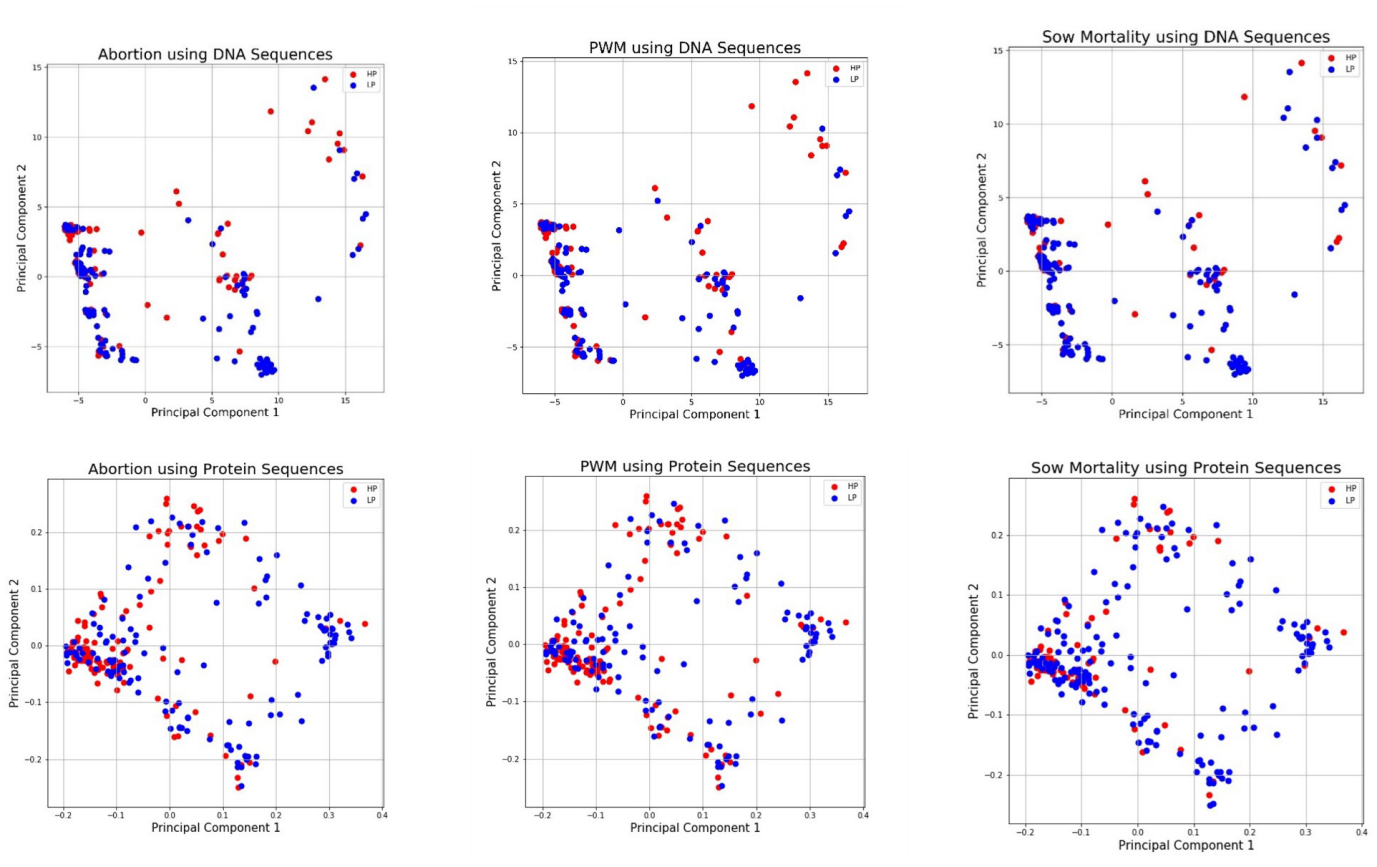
Low Dimensional PCA Visualization of DNA and Protein Sequences for Abortion (Top Left and Bottom Left), PWM (Middle Top and Bottom), and Sow Mortality (Top Right and Bottom Right) ^*^DNA, deoxyribose nucleic acid; PWM, pre-weaning mortality.

As nucleotides and amino acids are qualitative multivariate data with many categories, numerical conversion is required for estimating relationships among sequences quantitatively (47). Conventionally, dendrograms were used to analyze such relationships, but they are not very informative (47), at least for clinical impact classification. The lower-dimensional representation of DNA and amino acid sequences for each clinical outcome was obtained using PCA, and the PCA plots are shown in Figure 1. Each principal component axis shows specific connections and the distribution of underlying biological information present with fewer variables. From Figure 1, the X and Y axes of the PCA plot depict principal component 1 and principal component 2, respectively, which did not separate the samples into major grouping/clusters. Instead, we observed a high degree of class overlap between HI and LI classes for all three clinical signs used in the study.

## 4. Discussion

This study is an extension of the work done by Rosendal et al. (33) using machine learning approaches. Rosendal et al. initially collected the data and performed a study using the approaches described in (33) to identify the associations between different PRRSV RFLP types and the observed clinical signs. In the current study, we analyzed the high-dimensional ORF-5 region and herd-level demographic data for phenotypic mapping with the three clinical signs frequently of concern during PRRS outbreaks.

In general, *k*-mer count-based input representations were most informative in linking genotype with clinical impact for abortion and PWM using DNA and amino acid sequences, respectively. *K*-mers are alignment-free methods, and the large length *k*-mers showed discriminatory patterns between the LI/HI classes, which suggests that the distribution of *k*-mers was different for the LI/HI classes. However, a one-to-one correspondence for linking genotype–phenotype cannot be established. For *k*-mer input representations, with an increase in the value of *k*, the input becomes highly dimensional, which, when coupled with the small sample size inputs for training, results in a lack of statistical power for the predictive methods. However, alternative explanations are possible as varying sizes of *k*-mers (e.g., short versus long) yielded different performances for differentiating the species and host class distribution of different viruses (48). Regarding the size of the *k*-mer composition, it is too early to make strong conclusions about the clinical impact of PRRS, especially under field conditions that are subject to additional uncertainty and measurement errors in the outcome assessment.

The PCA feature extraction technique was intended to bring improvements to overcome the challenges of high-dimensional inputs by using dummy variables and *k*-mer representations. PCA removes noise, and the low dimensional embedded representations contain important extracted features and meaningful information, but it did not bring substantial improvement over other representations in predictive performance on the small PRRS dataset. A limitation of PCA is that it assumes a linear relationship between the input features, which is not always the case for real-world datasets. In our opinion, the underlying relationship between the input features of PRRS may be non-linear. Therefore, PCA representations may not capture the data’s true structure, resulting in low performance. In addition, these data are population-level disease impact estimates obtained through an observational study. As such, other confounders, measured and unmeasured, and a misclassification error of the outcome might have impacted this study in general and the PCA representation itself. Also, PCA helped visualize genetic sequences with a low-dimensional representation. PCA plots showed high overlap in the corresponding axes, which plausibly indicated the presence of shared motifs in the LI/HI classes. However, such overlap does not conclude the genetic relationship but gives a different understanding of the low performance of linear and non-linear baseline classifiers on the small PRRS dataset.

Therefore, with the preliminary evaluation of baseline classifiers presented in this study, we quantified the plausible extent of linkage by using different representations of the genetic and demographic datasets for abortion, PWM, and SM. Based on the limited dataset offered to ML algorithms in this study, the trend of achieving improvements in predictive performance over the baseline accuracy was consistently established for abortion and PWM. Such a trend indicates a plausible genotypic-phenotypic relationship for abortion, and PWM, which can be moderately established for PRRS in sow herds in Ontario. However, any plausible relationship could not be established with high confidence by using any of the input representations for SM. These results are concordant with the results of a recent study by Melmer, O’Sullivan, et al. (8). However, Melmer, O’Sullivan, et al. (8) used a different study population, and samples were collected in different periods. Melmer, O’Sullivan, et al. (8) investigated similar objectives through an approach that dealt with abortion, PWM, and SM on a quantitative rather than a discrete scale, using only the random forest model. Among the demographic, dummy, *k*-mer, and PCA-based input representations used in this study, the qualitative agreement in the results obtained from the two studies is that the best predictive results were achieved for assessing PWM, followed by abortion, with relatively little/no improvement for SM. The data collected by Rosendal et al. (33) were collected by retrospective telephone interviews and, therefore, subject to recall bias. Although there could be an underlying link between the composition of the PRRSV genome and the severity of specific clinical signs, the accuracy of reporting reproductive and respiratory clinical signs during PRRS outbreaks could also contribute to measurement error. This may be of particular relevance for SM, which could have multiple causes and has been a topic of attention to measurement and reporting, variability among herds, and temporal trends (49–51). In addition, a highly imbalanced class distribution was used to train the ML classifiers with a small sample size for SM, which is why the ML classifiers always ultimately favored the prediction of the majority class (i.e., the LI class).

Different classifiers showed improvement with different input data for the clinical outcomes used in this study. From a big-picture perspective, improvement in the predictive performance of best-performing individual classifiers over the baseline frequency of the majority class is approximately 17.31 and 18.65% in the case of abortion and PWM, respectively. Considering the complex nature of PRRS pathogenicity classification, adopting an integrative approach for studying the clinical impact classification of PRRSV strains using ensemble approaches is desirable. The consensus voting ensemble approach integrated different input datasets with varying input representations. Given the diverse number of classifiers, input dataset representations, and small sample size used in this study, the consensus voting approach effectively introduced a diversity of input representation, and the aggregating approach made the ensemble more stable. The consensus voting approach improved the predictive performance metrics by approximately 17.71% for abortion and 16.17% for PWM over baseline accuracy. In the case of small-sized datasets with moderately high variance for non-weak baseline classifiers, using the consensus voting approach may increase the stability of ensemble methods. However, using the consensus voting approach might not improve the performance metrics for the PRRS dataset and may benefit from a bigger genotypic-phenotypic input dataset.

## 5. Conclusion

Despite obvious clinical rationale, studies utilizing ML for PRRSV clinical impact classification continue to be rare. One reason for the lack of such studies is the limited availability of PRRS phenotypic information in the public domain. This study showed a linkage between the high impact of abortion and PWM with ORF-5 genetic sequences. Though demographic and management factors are known to play a role in the duration of PRRS outbreaks (52), this study could not establish the link. Measurement error in collecting demographic details for such phenotypic datasets always poses a risk which might be a reason for low improvements over baseline using herd-level demographic data. The improvements in results obtained from baseline classifiers and ensemble methods also resonate with the recent study by Melmer, O’Sullivan, et al. (8), adding consistency to obtained results using ML techniques for the three clinical signs used in this study. Though the accuracy was not high, our study made good progress in linking the ORF-5 genotype with phenotypic information using data-driven computational approaches.

Also, the integration of multiple representations of genetic data and herd-level demographic data presented in this work has not yet been considered in the literature for analyzing the impact of circulating strains in swine populations. The consensus voting ensemble approach used in this study could be extended on additional training data to assist veterinarians and domain experts in analyzing the currently circulating strains along with herd-level demographic details for the prognostic clinical impact analysis. However, further study with additional training data might help determine the genotypic-phenotypic relation for SM.

Our study has been largely successful in achieving the determined research objectives. The study suggests exploring other factors that could contribute to PRRS’s clinical impact, including other regions in the PRRSV genome, herd demographics, herd management factors, herd immunity status, and host genetics. Such an integrative approach for studying the clinical impact classification of PRRSV strains may be more informative in the absence of large genotypic datasets.

## Data availability statement

The data analyzed in this study is subject to the following licenses/restrictions: Due to privacy agreements, the genetic data cannot be shared publicly. Requests to access these datasets should be directed to zpoljak@uoguelph.ca.

## Ethics statement

The study was solely computational, and the dataset was obtained using a previous study where human participation was reviewed and approved by the Ethics Committee at the University of Guelph, Ontario, Canada. The participant herd owners provided their written informed consent to participate in this study.

## Author contributions

AC: conception, designed research, conducted research, analyzed and interpreted data, wrote and edited the paper, and reviewed the manuscript critically. ZP: analyzed and interpreted data, edited the paper, and reviewed the manuscript critically. RD, DP, and DG: study overview, review, and manuscript edits. TR: data collection and review of the manuscript. All authors contributed to the article and approved the submitted version.

## Funding

This study was supported by the Ontario Agri-Food Innovation Alliance, Food and Rural Affairs (OMAFRA) and the Natural Sciences and Engineering Research Council of Canada (NSERC) Discovery grant for their financial support of this project.

## Conflict of interest

The authors declare that the research was conducted in the absence of any commercial or financial relationships that could be construed as a potential conflict of interest.

## Publisher’s note

All claims expressed in this article are solely those of the authors and do not necessarily represent those of their affiliated organizations, or those of the publisher, the editors and the reviewers. Any product that may be evaluated in this article, or claim that may be made by its manufacturer, is not guaranteed or endorsed by the publisher.

## Footnotes

^1^https://www.geneious.com
