# Supplementary material for: Classification of porcine reproductive and respiratory syndrome clinical impact in Ontario sow herds using machine learning approaches
Source: Front Vet Sci. 2023 Jun 7;10:1175569. doi: 10.3389/fvets.2023.1175569 (PMC10284593; doi:10.3389/fvets.2023.1175569)
Supplement: Supplementary file 1 [file Data_Sheet_1.docx]

Supplementary Material

# Supplementary Section S1: Machine Learning Techniques for Sequence Classification and Use of Ensemble Methods

With a considerable increase in the collection of genomic sequences, data mining, machine learning (ML), and deep learning techniques gained momentum for data-driven analysis using genomic sequences (Inza *et al.*, 2010). When trained with sufficiently large datasets, ML techniques can learn from existing knowledge, with the predictive performance expected to be high with unseen data (Marsland, 2014). The ML techniques have been categorized into supervised, unsupervised, and semi-supervised. Classification is a form of supervised machine learning where input and output labels are used to train the predictive classifiers. Classification of DNA, amino acid sequences, and several other biological problems have been performed using ML techniques in previous studies (Middendorf *et al.*, 2004; Kundaje *et al.*, 2006; Zhang and Rajapakse, 2008; Inza *et al.*, 2010; Schaub *et al.*, 2012; Lanchantin *et al.*, 2017; Diao, Kohane and Manrai, 2018; Gupta and Kundaje, 2019). The classification of genetic sequences was further categorized into three broad categories: feature-based, distance-based, and model-based classification techniques (Xing, Pei, and Keogh, 2010). As no single classification technique works best for each classification problem, there needs to be an evaluation of a few classifiers from each category. Furthermore, as genomic sequence input is generally high dimensional, many ML classifiers suffer from the “curse of dimensionality” problem when trained with small-sampled datasets.

Classification methods like logistic regression (LR)with ridge and lasso regularization, decision tree (DT), random forest (RF), k nearest neighbor (KNN), and support vector machine (SVM)have been used for various classification purposes, including bioinformatics (Zhang and Rajapakse, 2008; Inza *et al.*, 2010; Fernández-Delgado *et al.*, 2014; Marsland, 2014). Logistic regression employs a logistic function to use input parameters for dichotomous classification purposes using maximum likelihood estimation (Huang, Liu, and Liang, 2016). Ridge and lasso regularization methods with LR stabilize the coefficients learned from the dataset (Huang, Liu, and Liang, 2016). A detailed introduction to LR can be found in (Motrenko, Strijov and Weber, 2014; Sperandei, 2014). RF is an ensemble approach in which the outcome of each independent decision tree is used to improve the generalization performance and bring stabilization in DTs (Breiman, 2001; Chen and Ishwaran, 2012; Qi, 2012). A detailed introduction to DT and RF can be found in (Salzberg *et al.*, 1998; Breiman, 2001; Qi, 2012; Song and Lu, 2015; Mantas *et al.*, 2019). KNN is a ‘lazy’ classification technique that assigns the majority label of K’s nearest neighbors to the test input (Kouiroukidis and Evangelidis, 2011). A detailed introduction to KNN can be found in (Kouiroukidis and Evangelidis, 2011; Xing and Bei, 2020; Zhang, 2021). Finally, SVM is a maximum margin hyperplane that uses a kernel trick. The linear kernel method uses linearly separable inputs by exploring a maximum margin hyperplane. Non-linear kernels are preferable for non-linear input datasets that reduce generalization errors (Rakotomamonjy, 2003; Vidovic *et al.*, 2015). A detailed introduction to SVM can be found in (Sonnenburg, Rätsch and Schölkopf, 2005; Rätsch, Sonnenburg and Schäfer, 2006; Wilson, 2008; Awad *et al.*, 2015; Ladwani, 2018; Chauhan, Dahiya and Sharma, 2019; Chandra and Bedi, 2021). When trained with sufficiently large datasets, deep learning classifiers can also learn non-linear dependencies present in the dataset(Chen and Lin, 2014; Lecun, Bengio and Hinton, 2015; Eraslan *et al.*, 2019).

Feature selection methods like forward feature selection and backward feature selection are used to select important input data features(Ang *et al.*, 2016; Li *et al.*, 2017; Bolón-Canedo and Alonso-Betanzos, 2018). Feature selection is used to reduce the high dimensionality of the input. The principal component analysis reduces input data dimensionality by a linear transformation. The low dimensional embedding contains important patterns observed from the data without losing essential information, and it can be further used as an input to the ML classifiers (Smith, 1988; Bi *et al.*, 2003; Kouiroukidis and Evangelidis, 2011).

Ensemble methods are a collection of learning classifiers that utilize decisions obtained from multiple classifiers using different features and datasets for making a prediction. It has been observed in the literature that the use of ensemble methods introduces diversity and flexibility, which further improves the generalization capabilities of the ensemble over individual classifiers (Chen, Wang, and Zhang, 2011; Chen and Ishwaran, 2012; Galar et al., 2012; Purushotham et al., 2018). Despite being computationally expensive, ensemble methods were frequently used in many competitive challenges, including the Netflix prize challenge (Koren, Bell, and Volinsky, 2009) and the ImageNet challenge (Chen and Ishwaran, 2012; Szegedy *et al.*, 2015; Hou *et al.*, 2019). In addition, different ensemble learning techniques like bootstrap aggregating/bagging, boosting, the bucket of models, and stacking have been used in bioinformatics applications (Inza *et al.*, 2010; Chen and Ishwaran, 2012; Galar *et al.*, 2012). Each ensemble model is assigned an equal weight in the bootstrap aggregating or bagging ensemble approach. Bagging is an ensemble meta-estimator that reduces the variance and results in a more accurate classification method than the different base classifiers used to build them (Opitz and Maclin, 1999). The bagging ensemble approach is also practical for unstable learning algorithms (Opitz and Maclin, 1999). Boosting-based ensemble methods incrementally build models to account for previously misclassified data points (Fernández-Delgado *et al.*, 2014). The boosting approach was primarily found to be more effective with weak learners but may/may not reduce the errors (Opitz and Maclin, 1999). The boosting technique helps reduce bias and improve the performance of existing classifiers. The voting ensemble combines the predictions from different classifiers. Consensus and majority voting ensemble techniques were used to overcome the problems of noisy datasets (Muhlenbach, Lallich, and Zighed, 2004; Galar et al., 2012). When the base classifiers used in ensemble experiments do not perform equally well, weighted voting improves the classification performance (Dogan and Birant, 2019). The stacking ensemble method combines a stack of classifiers to predict better (Liang *et al.*, 2020).

Opitz et al. (1999) have discussed that an effective combination of disagreed classifiers could reduce the expected error. Ensemble methods may also be used to tackle the problem of class imbalance (Galar *et al.*, 2012). Considering diversity among the component models as part of ensemble building helps to undermine the misclassifications using ensembles (Galar *et al.*, 2012; Zhou, 2012). Researchers have discussed the usage of small data samples for building models which generalize on large datasets (Seni and Elder, 2010; Kordík, Černý and Frýda, 2018).

**References**

Ang, J.C. *et al.* (2016)‘Supervised, unsupervised, and semi-supervised feature selection: A review on gene selection’, *IEEE/ACM Transactions on Computational Biology and Bioinformatics*, 13(5), pp. 971–989. Available at: https://doi.org/10.1109/TCBB.2015.2478454.

Awad, M. *et al.* (2015)‘Support Vector Machines for Classification’, in *Efficient Learning Machines*, pp. 39–66. Available at: https://doi.org/10.1007/978-1-4302-5990-9_3.

Bi, J. *et al.* (2003)‘Dimensionality reduction via sparse support vector machines’, *Journal of Machine Learning Research*, 3, pp. 1229–1243.

Bolón-Canedo, V. and Alonso-Betanzos, A. (2018)‘Feature selection’, in *Intelligent Systems Reference Library*, pp. 13–37. Available at: https://doi.org/10.1007/978-3-319-90080-3_2.

Breiman, L. (2001)‘Random forests’, *Machine Learning*, 45(1), pp. 5–32. Available at: https://doi.org/10.1023/A:1010933404324.

Chandra, M.A. and Bedi, S.S. (2021)‘Survey on SVM and their application in image classification’, *International Journal of Information Technology (Singapore)*, 13(5), pp. 1–11. Available at: https://doi.org/10.1007/s41870-017-0080-1.

Chauhan, V.K., Dahiya, K. and Sharma, A. (2019)‘Problem formulations and solvers in linear SVM: a review’, *Artificial Intelligence Review*, pp. 803–855. Available at: https://doi.org/10.1007/s10462-018-9614-6.

Chen, X. and Ishwaran, H. (2012)‘Random forests for genomic data analysis’, *Genomics*, pp. 323–329. Available at: https://doi.org/10.1016/j.ygeno.2012.04.003.

Chen, X., Wang, M. and Zhang, H. (2011)‘The use of classification trees for bioinformatics’, *Wiley Interdisciplinary Reviews: Data Mining and Knowledge Discovery*, 1(1), pp. 55–63. Available at: https://doi.org/10.1002/widm.14.

Chen, X.W. and Lin, X. (2014)‘Big data deep learning: Challenges and perspectives’, *IEEE Access*, pp. 514–525. Available at: https://doi.org/10.1109/ACCESS.2014.2325029.

Diao, J.A., Kohane, I.S. and Manrai, A.K. (2018)‘Biomedical informatics and machine learning for clinical genomics’, *Human Molecular Genetics*, pp. R29–R34. Available at: https://doi.org/10.1093/hmg/ddy088.

Dogan, A. and Birant, D. (2019)‘A Weighted Majority Voting Ensemble Approach for Classification’, in *UBMK 2019 - Proceedings, 4th International Conference on Computer Science and Engineering*, pp. 1–6. Available at: https://doi.org/10.1109/UBMK.2019.8907028.

Eraslan, G. *et al.* (2019)‘Deep learning: new computational modelling techniques for genomics’, *Nature Reviews Genetics*, pp. 389–403. Available at: https://doi.org/10.1038/s41576-019-0122-6.

Fernández-Delgado, M. *et al.* (2014)‘Do we need hundreds of classifiers to solve real world classification problems?’, *Journal of Machine Learning Research*, 15, pp. 3133–3181. Available at: https://doi.org/10.1117/1.JRS.11.015020.

Galar, M. *et al.* (2012)‘A review on ensembles for the class imbalance problem: Bagging-, boosting-, and hybrid-based approaches’, *IEEE Transactions on Systems, Man and Cybernetics Part C: Applications and Reviews*, pp. 463–484. Available at: https://doi.org/10.1109/TSMCC.2011.2161285.

Gupta, A. and Kundaje, A. (2019)‘Targeted optimization of regulatory DNA sequences with neural editing architectures’, *bioRxiv* [Preprint]. Available at: https://doi.org/10.1101/714402.

Hou, W. *et al.* (2019)‘An Advanced k Nearest Neighbor Classification Algorithm Based on KD-tree’, in *Proceedings of 2018 IEEE International Conference of Safety Produce Informatization, IICSPI 2018*, pp. 902–905. Available at: https://doi.org/10.1109/IICSPI.2018.8690508.

Huang, H.H., Liu, X.Y. and Liang, Y. (2016)‘Feature selection and cancer classification via sparse logistic regression with the hybrid L1/2 +2 regularization’, *PLoS ONE*, 11(5). Available at: https://doi.org/10.1371/journal.pone.0149675.

Inza, I. *et al.* (2010)‘Machine learning: an indispensable tool in bioinformatics.’, *Methods in molecular biology (Clifton, N.J.)*, 593, pp. 25–48. Available at: https://doi.org/10.1007/978-1-60327-194-3_2.

Kordík, P., Černý, J. and Frýda, T. (2018)‘Discovering predictive ensembles for transfer learning and meta-learning’, *Machine Learning*, 107(1), pp. 177–207. Available at: https://doi.org/10.1007/s10994-017-5682-0.

Koren, Y., Bell, R. and Volinsky, C. (2009)‘Matrix factorization techniques for recommender systems’, *Computer*, 42(8), pp. 30–37. Available at: https://doi.org/10.1109/MC.2009.263.

Kouiroukidis, N. and Evangelidis, G. (2011)‘The effects of dimensionality curse in high dimensional knn search’, in *Proceedings - 2011 Panhellenic Conference on Informatics, PCI 2011*, pp. 41–45. Available at: https://doi.org/10.1109/PCI.2011.45.

Kundaje, A. *et al.* (2006)‘A classification-based framework for predicting and analyzing gene regulatory response’, *BMC Bioinformatics*, 7(SUPPL.1), pp. 1–14. Available at: https://doi.org/10.1186/1471-2105-7-S1-S5.

Ladwani, V.M. (2018)*Support vector machines and applications*, *Computer Vision: Concepts, Methodologies, Tools, and Applications*. Available at: https://doi.org/10.4018/978-1-5225-5204-8.ch057.

Lanchantin, J. *et al.* (2017)‘Deep motif dashboard: visualizing and understanding genomic sequences using deep neural networks’, in *Pacific Symposium on Biocomputing*, pp. 254–265. Available at: https://doi.org/10.1142/9789813207813_0025.

Lecun, Y., Bengio, Y. and Hinton, G. (2015)‘Deep learning’, *Nature*, pp. 436–444. Available at: https://doi.org/10.1038/nature14539.

Li, J. *et al.* (2017)‘Feature selection: A data perspective’, *ACM Computing Surveys*, pp. 1–45. Available at: https://doi.org/10.1145/3136625.

Liang, D. *et al.* (2020)‘Combining corporate governance indicators with stacking ensembles for financial distress prediction’, *Journal of Business Research*, 120, pp. 137–146. Available at: https://doi.org/10.1016/j.jbusres.2020.07.052.

Mantas, CJ *et al.* (2019)‘A comparison of random forest based algorithms: random credal random forest versus oblique random forest’, *Soft Computing*, 23(21), pp. 10739–10754. Available at: https://doi.org/10.1007/s00500-018-3628-5.

Marsland, S. (2014)*Machine learning: An algorithmic perspective*, *Machine Learning: An Algorithmic Perspective, Second Edition*. Available at: https://doi.org/10.1201/b17476.

Middendorf, M. *et al.* (2004)‘Predicting genetic regulatory response using classification’, in *Bioinformatics*. Available at: https://doi.org/10.1093/bioinformatics/bth923.

Motrenko, A., Strijov, V. and Weber, G.W. (2014)‘Sample size determination for logistic regression’, *Journal of Computational and Applied Mathematics*, 255, pp. 743–752. Available at: https://doi.org/10.1016/j.cam.2013.06.031.

Muhlenbach, F., Lallich, S. and Zighed, D.A. (2004)‘Identifying and Handling Mislabelled Instances’, in *Journal of Intelligent Information Systems*, pp. 89–109. Available at: https://doi.org/10.1023/A:1025832930864.

Opitz, D. and Maclin, R. (1999)‘Popular Ensemble Methods: An Empirical Study’, *Journal of Artificial Intelligence Research*, 11, pp. 169–198. Available at: https://doi.org/10.1613/jair.614.

Purushotham, S. *et al.* (2018)‘Benchmarking deep learning models on large healthcare datasets’, *Journal of Biomedical Informatics*, 83, pp. 112–134. Available at: https://doi.org/10.1016/j.jbi.2018.04.007.

Qi, Y. (2012)‘Random forest for bioinformatics’, in *Ensemble Machine Learning: Methods and ApplicatiOns*, pp. 307–323. Available at: https://doi.org/10.1007/9781441993267_10.

Rakotomamonjy, A. (2003)‘Variable selection using SVM-based criteria’, *Journal of Machine Learning Research*, 3, pp. 1357–1370.

Rätsch, G., Sonnenburg, S. and Schäfer, C. (2006)‘Learning interpretable SVMs for biological sequence classification’, *BMC Bioinformatics*, 7(SUPPL.1). Available at: https://doi.org/10.1186/1471-2105-7-S1-S9.

Salzberg, S. *et al.* (1998)‘A decision tree system for finding genes in DNA’, *Journal of Computational Biology*, 5(4), pp. 667–680. Available at: https://doi.org/10.1089/cmb.1998.5.667.

Schaub, M.A. *et al.* (2012)‘Linking disease associations with regulatory information in the human genome’, *Genome Research*, 22(9), pp. 1748–1759. Available at: https://doi.org/10.1101/gr.136127.111.

Seni, G. and Elder, J.F. (2010)‘Ensemble Methods in Data Mining: Improving Accuracy Through Combining Predictions’, *Synthesis Lectures on Data Mining and Knowledge Discovery*, 2(1), pp. 1–126. Available at: https://doi.org/10.2200/s00240ed1v01y200912dmk002.

Smith, L. (1988)‘A tutorial on principal components analysis’, *Communications in Statistics - Theory and Methods*, 17(9), pp. 3157–3175. Available at: http://www.mendeley.com/research/computational-genome-analysis-an-introduction-statistics-for-biology-and-health/%5Cnhttp://www.tandfonline.com/doi/abs/10.1080/03610928808829796.

Song, YY and Lu, Y. (2015)‘Decision tree methods: applications for classification and prediction’, *Shanghai Archives of Psychiatry*, 27(2). Available at: https://doi.org/10.11919/j.issn.1002-0829.215044.

Sonnenburg, S., Rätsch, G. and Schölkopf, B. (2005)‘Large scale genomic sequence SVM classifiers’, in *ICML 2005 - Proceedings of the 22nd International Conference on Machine Learning*, pp. 849–856. Available at: https://doi.org/10.1145/1102351.1102458.

Sperandei, S. (2014)‘Understanding logistic regression analysis’, *Biochemia Medica*, 24(1), pp. 12–18. Available at: https://doi.org/10.11613/BM.2014.003.

Szegedy, C. *et al.* (2015)‘Going deeper with convolutions’, *Proceedings of the IEEE Computer Society Conference on Computer Vision and Pattern Recognition*, 07-12-June, pp. 1–9. Available at: https://doi.org/10.1109/CVPR.2015.7298594.

Vidovic, M.M.C. *et al.* (2015)‘SVM2Motif-reconstructing overlapping DNA sequence motifs by mimicking an SVM predictor’, *PLoS ONE*, 10(12). Available at: https://doi.org/10.1371/journal.pone.0144782.

Wilson, M.D. (2008)‘Support Vector Machines’, in *Encyclopedia of Ecology, Five-Volume Set*, pp. 3431–3437. Available at: https://doi.org/10.1016/B978-008045405-4.00168-3.

Xing, W. and Bei, Y. (2020)‘Medical Health Big Data Classification Based on KNN Classification Algorithm’, *IEEE Access*, 8, pp. 28808–28819. Available at: https://doi.org/10.1109/ACCESS.2019.2955754.

Xing, Z., Pei, J. and Keogh, E. (2010)‘A brief survey on sequence classification’, *ACM SIGKDD Explorations Newsletter*, 12(1), pp. 40–48. Available at: https://doi.org/10.1145/1882471.1882478.

Zhang, S. (2021)‘Challenges in KNN Classification’, *IEEE Transactions on Knowledge and Data Engineering*, pp. 4663–4675. Available at: https://doi.org/10.1109/TKDE.2021.3049250.

Zhang, Y.Q. and Rajapakse, J.C. (2008)*Machine learning in bioinformatics*, *Machine Learning in Bioinformatics*. Available at: https://doi.org/10.1002/9780470397428.

Zhou, Z.H. (2012)*Ensemble methods: Foundations and algorithms*, *Ensemble Methods: Foundations and Algorithms*. Available at: https://doi.org/10.1201/b12207.


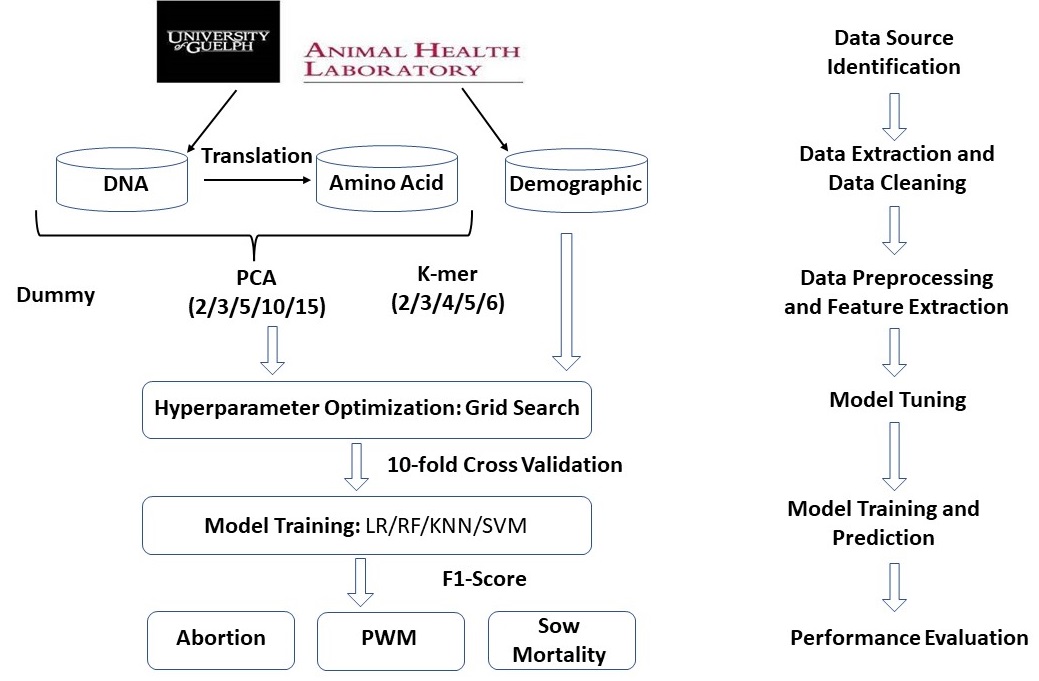


**Figure S1:** Proposed Approach for Pathogenicity Classification of PRRSV

* DNA: deoxyribose nucleic acid; PCA: principal component analysis; LR: logistic regression; RF: random forest; KNN: k nearest neighbor; SVM: support vector machine; PWM: pre-weaning mortality

* Image Reference: <https://www.uoguelph.ca/ahl/news?page=4>

**
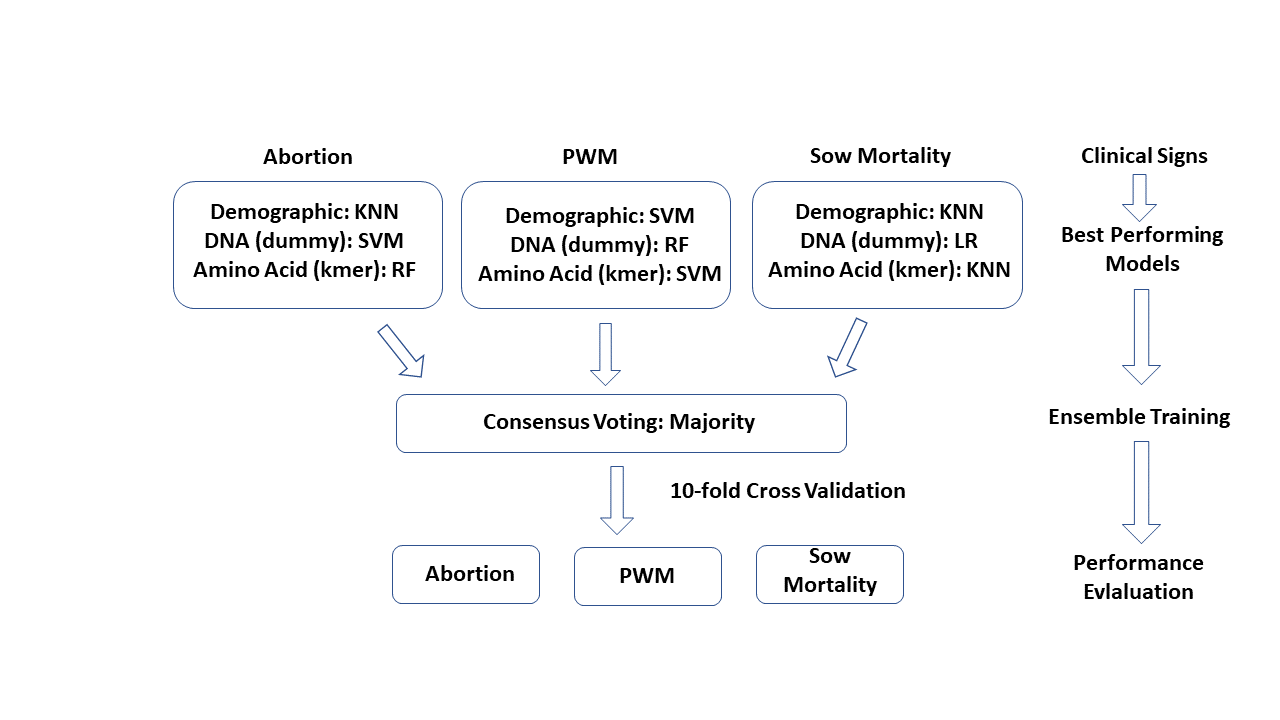
**

**Figure S2:** Consensus Voting Approach for Pathogenicity Classification of PRRSV

* DNA: deoxyribose nucleic acid; LR: logistic regression; RF: random forest; KNN: k nearest neighbor; SVM: support vector machine; PWM: pre-weaning mortality

**Supplementary Table S1:** Demographic Variables Used for Model Development

| **Variable** | **Type** | **Population Statistics/Distribution** | |
| --- | --- | --- | --- |
| **Number of sows in inventory** | Integer | Min-max | (35, 3000) |
|  |  | Mean | 539.67 |
| **Modified live vaccine (MLV)** | Binary | 0 (non-vaccinated) | 122 |
|  |  | 1 (vaccinated) | 125 |
| **Live virus inoculation (LVI)** | Binary | 0 (non-inoculation) | 224 |
|  |  | 1 (inoculation) | 23 |
| **Month of outbreak** | Categorical | January | 28 |
|  |  | February | 30 |
|  |  | March | 28 |
|  |  | April | 26 |
|  |  | May | 25 |
|  |  | June | 17 |
|  |  | July | 15 |
|  |  | August | 9 |
|  |  | September | 9 |
|  |  | October | 13 |
|  |  | November | 25 |
|  |  | December | 22 |
| **Type of herd** | Categorical | farrow-finish | 134 |
|  |  | farrow-wean | 68 |
|  |  | farrow-grow | 45 |

**Supplementary Table S2:** Hyper Parameters Used for Model Development

| **Algorithm** | **Hyper-parameters** | **Description** | **Values** |
| --- | --- | --- | --- |
| **LR**  **L1 (Lasso)**  **L2 (Ridge)** | Penalty | Regularization technique used | L1, L2 |
|  | C | The inverse of regularization strength | 0.5, 1.0, 1.5, 10, 50, …..100*** |
|  | solver | The algorithm used for the optimization | liblinear, SAGA |
|  | tol | Tolerance for stopping criteria | 0.0001, 0.001, 0.01, 0.1*** |
|  | max_iter | Maximum no. of iterations | 25, 50,….100 |
| **Random Forest** | criterion | The function to measure the quality of a split | Gini, Entropy |
|  | n_estimators | No. of trees in the forest | 10, 50, 100, 200*** |
|  | max_features | The max. number of features to consider for best splitting | 1, 2, 3, 4, 5, 10, 25, 50… 100, 500…... #max features*** |
|  | max_depth | Max. depth of the tree | 10, 20, 50 |
|  | max_leaf_nodes | Growing the tree in the best first fashion | 2, 45, 100 |
| **KNN (Euclidean)** | K | No. of neighbors | 3, 5, 7, 11, 15, 21 |
| **SVM (linear/RBF)** | C | Regularization parameter | 0.5, 1.0, 5.0, 10…..100*** |
|  | Kernel | Kernel type used in the algorithm | linear/RBF |

* Other parameters used as default values

** LR: logistic regression; RF: random forest; KNN: k nearest neighbor; SVM: support vector machine

*** The hyperparameter values used in each experiment depend on the input's data representation format and dimensionality. As for genomic sequences, the dimensionality largely depends on the input format. Also, some hyperparameter values depicted in the above Table may vary depending on the input format for the demographic data and genomic sequences (i.e., dummy, principal component, and k-mer representations of DNA and amino acid sequences may have different hyperparameter values used).

**Supplementary Table S3:** Results Obtained Using Demographic Dataset

| **Demographic** | **Abortion**  **(Baseline=52.22)** | | | | | | **PWM**  **(Baseline=50.60)** | | | | | | **Sow Mortality**  **(Baseline=70.04)** | | | | |
| --- | --- | --- | --- | --- | --- | --- | --- | --- | --- | --- | --- | --- | --- | --- | --- | --- | --- |
|  | **Acc** | **SN** | **SP** | **F1-score** | **AUC** |  | **Acc** | **SN** | **SP** | **F1-score** | **AUC** |  | **Acc** | **SN** | **SP** | **F1-score** | **AUC** |
| **LR** | 57.90,  (9.26) | 43.62, (17.18) | 71.36, (16.26) | 55.65, (10.26) | 60.26,  (13.81) |  | 55.47,  (4.54) | 60.49, (16.75) | 51.98, (19.29) | 53.90,  (5.02) | 57.23,  (8.22) |  | 68.95, (10.07) | 11.6,  (10.21) | 94.35,  (4.43) | 48.75,  (9.30) | 56.29,  (17.24) |
| **RF** | 54.60, (11.37) | 47.84, (15.51) | 63.02, (16.39) | 53.80, (11.77) | 60.68,  (12.64) |  | **57.03, (13.47)** | **62.18, (20.63)** | **55.57, (13.51)** | **56.87, (13.38)** | **61.58,**  **(12.10)** |  | 68.45, (11.62) | 1.25,  (3.95) | 97.06,  (9.30) | 41.45,  (5.72) | 53.36,  (20.17) |
| **KNN** | **60.65, (14.81)** | **49.21, (18.84)** | **72.39, (18.68)** | **59.40, (15.18)** | **59.35, (17.18)** |  | 53.43,  (8.73) | 67.10, (19.12) | 42.18, (12.62) | 51.97,  (8.72) | 55.86,  (8.54) |  | **68.85, (12.09)** | **16.58, (11.92)** | **91.97,**  **(9.62)** | **51.97, (11.50)** | **52.74,**  **(19.25)** |
| **SVM** | 60.27,  (13.40) | 46.12, (19.46) | 75.09, (14.12) | 58.73, (13.97) | 52.03,  (19.61) |  | 54.65,  (4.90) | 62.72, (15.71) | 49.28, (21.06) | 53.14,  (5.50) | 55.89,  (5.98) |  | 68.93,  (9.18) | 11.42, (13.01) | 94.53,  (5.18) | 48.26, (10.23) | 53.71,  (17.28) |

* The best-performing classifiers for each clinical sign are marked in bold in the above Table.

* LR: logistic regression; RF: random forest; KNN: k nearest neighbor; SVM: support vector machine; Acc: accuracy; SN: sensitivity, SP: specificity, AUC: area under the curve

**Supplementary Table S4:** Results Obtained Using Dummy Representation of DNA Sequences

| **Dummy DNA** | **Abortion**  **(Baseline=52.22)** | | | | | | **PWM**  **(Baseline=50.60)** | | | | | | **Sow Mortality**  **(Baseline=70.04)** | | | | |  |
| --- | --- | --- | --- | --- | --- | --- | --- | --- | --- | --- | --- | --- | --- | --- | --- | --- | --- | --- |
|  | **Acc** | **SN** | **SP** | **F1-score** | **AUC** |  | **Acc** | **SN** | **SP** | **F1-score** | **AUC** |  | **Acc** | **SN** | **SP** | **F1-score** | **AUC** | |
| **LR** | 65.07, (11.30) | 70.59, (13.57) | 61.41, (13.38) | 64.77, (11.29) | 69.52,  (10.78) |  | 60.75, (8.11) | 67.94, (13.88) | 55.06, (14.05) | 59.84, (7.95) | 64.07,  (7.77) |  | 66.88, (7.64) | 9.75, (10.76) | 92.16, (6.92) | 45.51, (5.94) | 60.4,  (12.35) | |
| **RF** | 65.92, (10.38) | 75.33, (13.78) | 56.24, (12.76) | 64.84, (10.13) | 69.08,  (9.46) |  | **61.97, (7.94)** | **66.83, (12.22)** | **58.76, (12.36)** | **61.37, (7.60)** | **68.95,**  **(6.57)** |  | 68.53, (10.43) | 2.92,  (6.23) | 96.59, (7.81) | 42.22, (4.96) | 60.56,  (11.32) | |
| **KNN** | 65.55, (9.67) | 68.75, (12.03) | 64.17, (12.81) | 65.27, (9.64) | 67.96,  (10.85) |  | 51.00, (6.15) | 54.10, (12.93) | 51.18, (12.88) | 50.63, (5.96) | 53.58,  (6.98) |  | **69.33, (10.35)** | **10.58, (20.71)** | **96.06, (4.52)** | **46.62, (11.80)** | **60.26,**  **(10.29)** | |
| **SVM** | **68.33, (9.76)** | **80.71, (12.68)** | **57.36, (11.07)** | **67.61, (9.77)** | **70.63,**  **(10.62)** |  | 59.17, (7.56) | 67.16, (16.59) | 51.45, (11.16) | 57.85, (7.01) | 64.36,  (10.67) |  | 70.13, (9.66) | 0.0,  (0.0) | 100.0, (0.0) | 41.05, (3.41) | 48.41,  (10.52) | |

* The best-performing classifiers for each clinical sign are marked in bold in the above Table.

* LR: logistic regression; RF: random forest; KNN: k nearest neighbor; SVM: support vector machine; Acc: accuracy; SN: sensitivity, SP: specificity, AUC: area under the curve

**Supplementary Table S5:** Results Obtained Using Dummy Representation of Amino Acid Sequences

| **Dummy**  **Protein** | **Abortion**  **(Baseline=52.22)** | | | | | | **PWM**  **(Baseline=50.60)** | | | | | **Sow Mortality**  **(Baseline=70.04)** | | | | | |
| --- | --- | --- | --- | --- | --- | --- | --- | --- | --- | --- | --- | --- | --- | --- | --- | --- | --- |
|  | **Acc** | **SN** | **SP** | **F1-score** | **AUC** |  | **Acc** | **SN** | **SP** | **F1-score** | **AUC** |  | **Acc** | **SN** | **SP** | **F1-score** | **AUC** |
| **LR** | 63.85, (9.27) | 63.86, (12.24) | 65.05, (11.96) | 63.46,  (9.22) | 66.78,  (9.56) |  | 62.00, (7.37) | 67.06, (13.28) | 57.09, (10.99) | 61.02, (7.27) | 65.18,  (11.12) |  | 64.40, (8.68) | 6.83, (12.33) | 89.97, (10.09) | 42.22, (7.00) | 57.39,  (11.31) |
| **RF** | 63.07, (11.39) | 69.23, (16.79) | 59.55, (13.01) | 62.81, (11.33) | 66.62,  (12.25) |  | 60.77, (5.69) | 69.18, (10.78) | 53.67, (14.69) | 59.66, (5.62) | 66.68,  (5.48) |  | 68.15, (11.63) | 5.42, (10.77) | 94.44, (6.38) | 43.95, (8.81) | 58.93,  (16.38) |
| **KNN** | 63.87, (11.06) | 71.98, (15.81) | 58.05, (10.65) | 63.60, (11.00) | 66.52,  (8.91) |  | 54.22, (5.67) | 68.73, (13.34) | 41.84, (14.24) | 52.55, (6.50) | 57.20,  (6.44) |  | **66.43, (5.80)** | **9.08, (11.50)** | **92.08, (8.21)** | **45.46, (7.53)** | **56.72,**  **(11.78)** |
| **SVM** | **64.67, (12.65)** | **72.67, (15.20)** | **57.94, (14.63)** | **64.10, (12.37)** | **68.25,**  **(11.17)** |  | **63.95, (9.58)** | **72.47, (14.35)** | **55.97, (10.77)** | **63.01, (9.28)** | **68.53,**  **(10.35)** |  | 70.13, (9.66) | 0.0,  (0.0) | 100.0, (0.0) | 41.05, (3.41) | 56.10,  (8.79) |

* The best-performing classifiers for each clinical sign are marked in bold in the above Table.

* LR: logistic regression; RF: random forest; KNN: k nearest neighbor; SVM: support vector machine; Acc: accuracy; SN: sensitivity, SP: specificity, AUC: area under the curve

**Supplementary Table S6:** Results Obtained Using Principal Component-Based Representation of DNA Sequences for Abortion (Baseline=52.22)

| **Classifier** | **LR** | | | | |  | **RF** | | | | | **KNN** | | | | | | **SVM** | | | | | |
| --- | --- | --- | --- | --- | --- | --- | --- | --- | --- | --- | --- | --- | --- | --- | --- | --- | --- | --- | --- | --- | --- | --- | --- |
| **Principal Components** | **Acc** | **SN** | **SP** | **F1-score** | **AUC** |  | **Acc** | **SN** | **SP** | **F1-score** | **AUC** |  | **Acc** | **SN** | **SP** | **F1-score** | **AUC** |  | **Acc** | **SN** | **SP** | **F1-score** | **AUC** |
| **PC=2** | 63.93, (8.53) | 73.58, (11.89) | 55.70, (14.04) | 63.19, (8.86) | 70.67,  (10.61) |  | 65.17, (10.46) | 82.32, (13.55) | 49.4, (9.56) | 64.02, (10.49) | 67.55,  (10.28) |  | **66.33, (11.23)** | **76.38, (12.15)** | **58.4, (15.46)** | **65.78, (11.07)** | **69.86,**  **(8.36)** |  | 63.87, (12.84) | 76.19, (14.43) | 53.45, (14.41) | 63.18, (13.11) | 69.52,  (10.90) |
| **PC =3** | 64.30, (8.48) | 75.89, (13.32) | 54.36, (12.08) | 63.52, (8.68) | 70.66,  (11.89) |  | **66.38, (10.2)** | **81.07, (15.95)** | **52.07, (9.61)** | **65.15, (10.60)** | **66.79,**  **(13.06)** |  | 65.97, (9.32) | 76.45, (15.28) | 55.89, (11.49) | 64.99, (9.34) | 67.81,  (9.82) |  | 67.12, (10.31) | 82.31, (11.36) | 53.36, (11.15) | 66.19, (10.40) | 70.36,  (11.51) |
| **PC=5** | 66.72, (10.42) | 74.64, (8.81) | 59.18, (15.25) | 65.94, (10.73) | 69.84,  (12.05) |  | 63.98, (11.32) | 76.30, (19.25) | 53.27, (8.69) | 63.25, (11.58) | 66.13,  (12.99) |  | 63.02, (11.60) | 71.21, (19.68) | 55.88, (12.10) | 62.33, (12.04) | 66.27,  (12.66) |  | 65.53, (11.38) | 77.26, (17.35) | 55.52, (11.01) | 64.81, (11.42) | 67.60,  (12.63) |
| **PC=10** | 65.07, (12.01) | 72.19, (17.24) | 59.03, (16.88) | 64.20, (12.28) | 68.02,  (11.25) |  | 65.2, (10.58) | 77.07, (19.09) | 54.70, (8.77) | 64.37, (10.92) | 66.25,  (12.29) |  | 62.30, (7.15) | 58.49, (13.05) | 67.10, (12.06) | 61.58, (7.19) | 65.90,  (9.09) |  | **68.33, (9.20)** | **80.83, (13.36)** | **57.43, (10.37)** | **67.60, (9.28)** | **69.32,**  **(10.73)** |
| **PC=15** | **67.95, (9.60)** | **76.78, (15.17)** | **60.02, (13.47)** | **67.16, (9.96)** | **68.59,**  **(9.43)** |  | 64.37, (12.39) | 77.55, (17.7) | 53.40, (10.9) | 63.78, (12.49) | 64.33,  (12.61) |  | 63.13, (12.67) | 62.90, (14.61) | 63.91, (17.22) | 62.60, (12.93) | 66.23,  (12.52) |  | 65.90, (10.28) | 76.86, (15.65) | 55.73, (11.64) | 65.01, (10.37) | 67.35,  (11.61) |
| **PC=20** | 65.52, (11.66) | 72.9, (15.46) | 59.26, (13.49) | 64.97, (11.76) | 67.55,  (13.06) |  | 63.17, (9.78) | 73.06, (15.87) | 55.14, (14.98) | 62.48, (9.87) | 64.10,  (10.53) |  | 61.50, (11.65) | 59.57, (12.93) | 64.65, (15.31) | 61.10, (11.47) | 67.14,  (12.84) |  | 63.50, (10.8) | 71.70, (15.69) | 56.73, (10.52) | 63.04, (10.89) | 65.11,  (10.37) |

* The best-performing classifiers for each clinical sign are marked in bold in the above Table.

* LR: logistic regression; RF: random forest; KNN: k nearest neighbor; SVM: support vector machine; Acc: accuracy; SN: sensitivity, SP: specificity, AUC: area under the curve

**Supplementary Table S7:** Results Obtained Using Principal Component-Based Representation of DNA Sequences for PWM (Baseline=50.60)

| **Classifier** | **LR** | | | | | | **RF** | | | | | **KNN** | | | | | | **SVM** | | | | | |
| --- | --- | --- | --- | --- | --- | --- | --- | --- | --- | --- | --- | --- | --- | --- | --- | --- | --- | --- | --- | --- | --- | --- | --- |
| **Principal Components** | **Acc** | **SN** | **SP** | **F1-score** | **AUC** |  | **Acc** | **SN** | **SP** | **F1-score** | **AUC** |  | **Acc** | **SN** | **SP** | **F1-score** | **AUC** |  | **Acc** | **SN** | **SP** | **F1-score** | **AUC** |
| **PC=2** | 58.22, (9.4) | 70.99, (18.49) | 44.71, (15.55) | 55.71, (8.27) | 62.45,  (10.41) |  | **58.27, (7.04)** | **68.16, (14.5)** | **49.09, (9.66)** | **57.11, (6.46)** | **61.55,**  **(11.35)** |  | **59.10, (4.04)** | **63.61, (16.37)** | **54.73, (15.67)** | **57.53, (4.02)** | **60.61,**  **(6.88)** |  | 57.45, (9.12) | 72.46, (13.18) | 44.13, (11.57) | 56.12, (9.00) | 63.51,  (10.91) |
| **PC =3** | **58.62, (10.45)** | **68.79, (18.02)** | **51.46, (13.82)** | **57.93, (10.15)** | **62.24,**  **(10.04)** |  | 55.87, (9.59) | 65.32, (15.02) | 47.21, (11.17) | 54.75, (8.81) | 60.03,  (11.96) |  | 55.07, (6.61) | 56.93, (13.49) | 53.98, (14.35) | 54.01, (5.53) | 59.76,  (5.71) |  | **61.08, (8.58)** | **77.30, (12.30)** | **44.84, (10.78)** | **59.26, (9.07)** | **63.13,**  **(10.02)** |
| **PC=5** | 57.42, (8.79) | 67.43, (18.1) | 50.21, (11.88) | 56.65, (8.42) | 62.26,  (10.22) |  | 58.28, (7.22) | 70.62, (14.18) | 46.59, (9.02) | 56.93, (6.60) | 61.80,  (8.23) |  | 54.97, (6.32) | 64.25, (15.43) | 46.72, (8.01) | 53.87, (6.53) | 53.86,  (5.23) |  | 57.50, (6.82) | 79.53, (11.64) | 36.41, (8.25) | 54.86, (6.06) | 61.95,  (11.04) |
| **PC=10** | 57.42, (8.79) | 67.43, (18.1) | 50.21, (11.88) | 56.65, (8.42) | 61.72,  (10.35) |  | 51.78, (7.45) | 57.51, (15.25) | 48.8, (13.23) | 51.12, (7.30) | 55.08,  (7.20) |  | 56.7, (9.79) | 57.46, (15.13) | 59.06, (20.02) | 55.93, (9.92) | 58.54,  (10.29) |  | 55.50, (7.59) | 77.48, (13.47) | 35.45, (10.54) | 52.89, (7.47) | 59.75,  (11.80) |
| **PC=15** | 57.42, (8.92) | 65.5, (19.1) | 51.87, (13.55) | 56.58, (8.44) | 63.07,  (9.62) |  | 52.23, (5.89) | 53.21, (15.29) | 54.02, (15.41) | 51.57, (6.42) | 54.98,  (5.96) |  | 51.83, (8.43) | 54.4, (16.02) | 48.06, (11.15) | 50.50, (8.26) | 53.27,  (7.32) |  | 57.50, (8.31) | 71.98, (13.5) | 42.61, (6.51) | 55.80, (7.89) | 63.01,  (12.56) |
| **PC=20** | 57.83, (8.41) | 65.53, (14.60) | 51.87, (11.96) | 57.05, (7.77) | 63.25,  (10.89) |  | 55.13, (6.71) | 60.12, (10.14) | 52.49, (12.83) | 54.65, (6.63) | 58.14,  (8.53) |  | 53.05, (7.74) | 53.65, (12.99) | 52.87, (13.11) | 52.17, (7.94) | 56.51,  (7.99) |  | 57.08, (7.87) | 69.83, (14.93) | 44.21, (8.57) | 55.50, (7.65) | 61.67,  (13.49) |

* The best-performing classifiers for each clinical sign are marked in bold in the above Table.

* LR: logistic regression; RF: random forest; KNN: k nearest neighbor; SVM: support vector machine; Acc: accuracy; SN: sensitivity, SP: specificity, PC: principal component, AUC: area under the curve

**Supplementary Table S8:** Results Obtained Using Principal Component-Based Representation of DNA Sequences for Sow Mortality (Baseline=70.04)

| **Classifier** | **LR** | | | | | | **RF** | | | | | **KNN** | | | | | | **SVM** | | | | | |
| --- | --- | --- | --- | --- | --- | --- | --- | --- | --- | --- | --- | --- | --- | --- | --- | --- | --- | --- | --- | --- | --- | --- | --- |
| **Principal Components** | **Acc** | **SN** | **SP** | **F1-score** | **AUC** |  | **Acc** | **SN** | **SP** | **F1-score** | **AUC** |  | **Acc** | **SN** | **SP** | **F1-score** | **AUC** |  | **Acc** | **SN** | **SP** | **F1-score** | **AUC** |
| **PC=2** | 68.5, (8.38) | 0.0, (0.0) | 97.92, (3.67) | 40.52, (3.02) | 61.85,  (9.81) |  | 70.13, (9.66) | 0.0, (0.0) | 100.0, (0.0) | 41.05, (3.41) | 64.08,  (11.08) |  | **66.47, (7.96)** | **13.33, (20.77)** | **91.41, (8.24)** | **46.37, (9.72)** | **59.97,**  **(14.46)** |  | 69.32, (9.06) | 0.0, (0.0) | 98.94, (2.26) | 40.78, (3.25) | 47.03,  (10.25) |
| **PC =3** | 68.92, (8.82) | 0.0, (0.0) | 98.41, (2.57) | 40.65, (3.17) | 62.04,  (12.90) |  | 70.13, (9.66) | 0.0, (0.0) | 100.0, (0.0) | 41.05, (3.41) | 62.10,  (11.13) |  | 66.47, (9.26) | 10.42, (12.31) | 91.71, (6.27) | 46.13, (9.89) | 60.73,  (9.41) |  | **70.13, (9.66)** | **0.0, (0.0)** | **100.0, (0.0)** | **41.05, (3.41)** | **55.04,**  **(19.23)** |
| **PC=5** | 68.52, (8.75) | 0.0, (0.0) | 97.94, (5.05) | 40.51, (3.14) | 63.88,  (12.63) |  | 70.13, (9.66) | 0.0, (0.0) | 100.0, (0.0) | 41.05, (3.41) | 60.19,  (12.39) |  | 68.48, (8.16) | 1.25, (3.95) | 97.38, (4.80) | 41.59, (4.90) | 56.89,  (11.36) |  | **70.13, (9.66)** | **0.0, (0.0)** | **100.0, (0.0)** | **41.05, (3.41)** | **50.48,**  **(11.48)** |
| **PC=10** | **68.92, (8.82)** | **1.25, (3.95)** | **97.88, (3.7)** | **41.72, (4.94)** | **63.42,**  **(15.68)** |  | 70.13, (9.66) | 0.0, (0.0) | 100.0, (0.0) | 41.05, (3.41) | 62.80,  (8.47) |  | 65.63, (6.05) | 9.58, (20.98) | 92.19, (10.0) | 43.08, (7.07) | 59.49,  (11.59) |  | **70.13, (9.66)** | **0.0, (0.0)** | **100.0, (0.0)** | **41.05, (3.41)** | **49.90,**  **(16.87)** |
| **PC=15** | 68.52, (8.75) | 0.0, (0.0) | 97.88, (3.70) | 40.51, (3.14) | 62.31,  (16.04) |  | 68.92, (9.26) | 0.0, (0.0) | 98.39, (3.59) | 40.64, (3.26) | 60.31,  (11.59) |  | 69.35, (10.32) | 2.67, (5.84) | 97.63, (4.14) | 42.92, (6.44) | 59.41,  (10.65) |  | **70.13, (9.66)** | **0.0, (0.0)** | **100.0, (0.0)** | **41.05, (3.41)** | **48.20,**  **(15.81)** |
| **PC=20** | 68.12, (8.87) | 0.0, (0.0) | 97.36, (5.13) | 40.37, (3.18) | 63.49,  (14.49) |  | **70.93, (9.23)** | **3.50, (8.18)** | **99.41, (1.86)** | **43.93, (6.50)** | **62.11,**  **(8.99)** |  | 70.13, (8.69) | 3.75, (6.35) | 98.33, (2.69) | 43.95, (5.21) | 57.90,  (11.40) |  | **70.13, (9.66)** | **0.0, (0.0)** | **100.0, (0.0)** | **41.05, (3.41)** | **47.94,**  **(12.31)** |

* The best-performing classifiers for each clinical sign are marked in bold in the above Table.

* LR: logistic regression; RF: random forest; KNN: k nearest neighbor; SVM: support vector machine; Acc: accuracy; SN: sensitivity, SP: specificity, PC: principal component, AUC: area under the curve

**Supplementary Table S9:** Results Obtained Using Principal Component-Based Representation of Amino Acid Sequences for Abortion (Baseline=52.22)

| **Classifier** | **LR** | | | | | | **RF** | | | | | **KNN** | | | | | | **SVM** | | | | | |
| --- | --- | --- | --- | --- | --- | --- | --- | --- | --- | --- | --- | --- | --- | --- | --- | --- | --- | --- | --- | --- | --- | --- | --- |
| **Principal Components** | **Acc** | **SN** | **SP** | **F1-score** | **AUC** |  | **Acc** | **SN** | **SP** | **F1-score** | **AUC** |  | **Acc** | **SN** | **SP** | **F1-score** | **AUC** |  | **Acc** | **SN** | **SP** | **F1-score** | **AUC** |
| **PC=2** | **68.73, (8.64)** | **79.93, (16.91)** | **58.24, (16.34)** | **67.46, (8.25)** | **70.24,**  **(10.79)** |  | 63.10, (10.80) | 70.60, (18.81) | 58.65, (11.85) | 62.70, (10.82) | 67.70,  (10.46) |  | **67.98, (9.32)** | **76.25, (16.92)** | **61.20, (11.89)** | **67.36, (9.37)** | **70.31,**  **(11.88)** |  | 66.73, (11.68) | 83.85, (11.91) | 51.45, (11.92) | 65.74, (11.60) | 70.54,  (11.04) |
| **PC =3** | 67.92, (8.95) | 79.99, (15.20) | 56.43, (12.96) | 66.79, (8.54) | 71.19,  (10.85) |  | 59.03, (8.14) | 55.29, (19.07) | 62.70, (18.10) | 57.38, (7.87) | 63.30,  (9.19) |  | 67.18, (8.61) | 74.15, (14.39) | 61.33, (12.86) | 66.47, (8.71) | 70.54,  (9.70) |  | 65.08, (13.05) | 78.02, (14.32) | 55.31, (14.16) | 64.67, (13.08) | 71.49,  (10.97) |
| **PC=5** | 65.92, (10.78) | 77.3, (16.85) | 55.76, (12.14) | 65.07, (10.33) | 70.15,  (10.68) |  | **65.85, (12.76)** | **67.13, (15.93)** | **65.98, (14.02)** | **65.46, (12.64)** | **69.40,**  **(14.34)** |  | 65.15, (9.64) | 69.38, (15.00) | 60.71, (9.87) | 64.40, (9.64) | 69.29,  (10.82) |  | 62.30, (14.30) | 71.40, (17.29) | 55.46, (14.59) | 61.81, (14.11) | 67.78,  (9.96) |
| **PC=10** | 67.52, (11.46) | 72.62, (17.29) | 63.12, (13.62) | 66.83, (11.53) | 68.68,  (10.58) |  | 63.88, (10.89) | 61.86, (12.87) | 65.94, (14.39) | 63.22, (10.79) | 64.61,  (11.10) |  | 63.95, (10.75) | 64.66, (16.34) | 63.88, (9.97) | 63.46, (11.15) | 65.07,  (10.37) |  | **68.70, (10.52)** | **77.48, (14.69)** | **62.01, (13.05)** | **68.25, (10.33)** | **68.48,**  **(9.34)** |
| **PC=15** | 64.3, (12.02) | 75.37, (17.19) | 54.21, (17.62) | 63.07, (12.30) | 67.90,  (10.59) |  | 58.27, (9.07) | 58.97, (21.76) | 60.02, (14.15) | 57.50, (9.24) | 64.07,  (10.70) |  | 66.78, (10.00) | 67.74, (15.06) | 67.07, (11.05) | 66.39, (10.16) | 67.09,  (8.53) |  | 65.08, (11.64) | 73.69, (16.78) | 57.64, (13.52) | 64.37, (11.51) | 67.91,  (9.75) |
| **PC=20** | 66.32, (12.56) | 72.14, (19.49) | 62.31, (11.63) | 65.85, (12.46) | 70.05,  (8.23) |  | 57.85, (12.77) | 65.23, (26.30) | 52.17, (10.39) | 56.79, (13.01) | 64.29,  (14.80) |  | 64.77, (12.30) | 60.88, (18.57) | 69.97, (15.11) | 63.94, (12.58) | 66.96,  (10.90) |  | 66.70, (11.37) | 73.99, (12.74) | 60.36, (14.74) | 66.06, (11.28) | 68.96,  (10.42) |

* The best-performing classifiers for each clinical sign are marked in bold in the above Table.

* LR: logistic regression; RF: random forest; KNN: k nearest neighbor; SVM: support vector machine; Acc: accuracy; SN: sensitivity, SP: specificity, PC: principal component, AUC: area under the curve

**Supplementary Table S10:** Results Obtained Using Principal Component-Based Representation of Amino Acid Sequences for PWM (Baseline=50.60)

| **Classifier** | **LR** | | | | | | **RF** | | | | | **KNN** | | | | | | **SVM** | | | | | |
| --- | --- | --- | --- | --- | --- | --- | --- | --- | --- | --- | --- | --- | --- | --- | --- | --- | --- | --- | --- | --- | --- | --- | --- |
| **Principal Components** | **Acc** | **SN** | **SP** | **F1-score** | **AUC** |  | **Acc** | **SN** | **SP** | **F1-score** | **AUC** |  | **Acc** | **SN** | **SP** | **F1-score** | **AUC** |  | **Acc** | **SN** | **SP** | **F1-score** | **AUC** |
| **PC=2** | 57.85, (7.42) | 70.98, (18.63) | 47.88, (12.44) | 56.77, (7.04) | 63.61,  (10.56) |  | 57.05, (7.94) | 73.51, (22.07) | 42.55, (14.79) | 54.7, (6.51) | 62.09,  (10.02) |  | 57.47, (7.34) | 65.63, (17.52) | 50.83, (14.87) | 56.13, (6.47) | 63.30,  (6.50) |  | 57.87, (8.70) | 77.40, (11.04) | 38.78, (10.09) | 55.58, (8.12) | 62.90,  (9.35) |
| **PC =3** | 58.65, (8.58) | 72.59, (18.56) | 47.88, (10.95) | 57.63, (8.19) | 63.0,  (10.37) |  | 55.43, (8.17) | 71.92, (24.75) | 43.80, (14.84) | 53.70, (7.84) | 58.47,  (11.65) |  | **57.43, (9.59)** | **67.22, (12.29)** | **49.6, (12.51)** | **56.71, (9.50)** | **57.86,**  **(9.40)** |  | 60.30, (5.81) | 77.36, (12.15) | 44.69, (7.31) | 58.77, (5.39) | 64.68,  (7.63) |
| **PC=5** | 59.08, (9.38) | 68.76, (15.94) | 51.1, (12.27) | 58.16, (9.05) | 65.17,  (10.17) |  | 57.90, (4.67) | 80.77, (11.36) | 36.49, (8.18) | 55.25, (4.42) | 67.37,  (9.14) |  | 55.05, (9.91) | 61.58, (13.86) | 50.78, (17.87) | 54.17, (9.63) | 56.88,  (7.30) |  | **62.78, (5.70)** | **82.57, (8.12)** | **43.03, (8.24)** | **60.52, (6.09)** | **63.96,**  **(11.55)** |
| **PC=10** | 59.88, (9.04) | 73.15, (16.80) | 46.92, (15.68) | 57.88, (8.49) | 63.56,  (10.81) |  | **58.32, (5.16)** | **76.18, (13.69)** | **41.84, (12.8)** | **56.23, (4.01)** | **61.01,**  **(4.71)** |  | 56.30, (10.84) | 61.17, (8.12) | 54.33, (20.85) | 55.72, (10.91) | 57.97,  (12.55) |  | 58.73, (7.13) | 80.77, (12.56) | 39.61, (11.32) | 56.63, (6.55) | 59.02,  (8.10) |
| **PC=15** | **61.50, (7.92)** | **70.19, (14.90)** | **55.21, (13.14)** | **60.74, (7.60)** | **66.20,**  **(9.93)** |  | 56.73, (9.01) | 64.71, (11.90) | 48.25, (14.19) | 55.37, (8.08) | 58.26,  (10.01) |  | 53.40, (10.60) | 57.95, (14.27) | 52.02, (18.92) | 52.90, (10.44) | 57.12,  (12.38) |  | 57.15, (8.87) | 74.28, (13.63) | 41.36, (11.36) | 55.35, (8.56) | 60.46,  (9.01) |
| **PC=20** | 61.08, (7.71) | 71.92, (15.36) | 52.21, (11.31) | 60.10, (7.47) | 67.34,  (9.50) |  | 56.70, (8.58) | 70.48, (18.76) | 44.83, (14.58) | 55.11, (8.42) | 59.11,  (11.22) |  | 56.68, (7.62) | 59.67, (10.62) | 56.13, (12.35) | 56.33, (7.57) | 61.09,  (8.43) |  | 56.30, (8.69) | 71.79, (15.39) | 42.47, (11.29) | 54.76, (8.01) | 62.80,  (10.81) |

* The best-performing classifiers for each clinical sign are marked in bold in the above Table.

* LR: logistic regression; RF: random forest; KNN: k nearest neighbor; SVM: support vector machine; Acc: accuracy; SN: sensitivity, SP: specificity, PC: principal component, AUC: area under the curve

**Supplementary Table S11:** Results Obtained Using Principal Component-Based Representation of Amino Acid Sequences for Sow Mortality (Baseline=70.04)

| **Classifier** | **LR** | | | | | | **RF** | | | | | **KNN** | | | | | | **SVM** | | | | | |
| --- | --- | --- | --- | --- | --- | --- | --- | --- | --- | --- | --- | --- | --- | --- | --- | --- | --- | --- | --- | --- | --- | --- | --- |
| **Principal Components** | **Acc** | **SN** | **SP** | **F1-score** | **AUC** |  | **Acc** | **SN** | **SP** | **F1-score** | **AUC** |  | **Acc** | **SN** | **SP** | **F1-score** | **AUC** |  | **Acc** | **SN** | **SP** | **F1-score** | **AUC** |
| **PC=2** | **70.13, (9.66)** | **0.0, (0.0)** | **100.0, (0.0)** | **41.05, (3.41)** | **63.26,**  **(10.07)** |  | 70.13, (9.66) | 0.0, (0.0) | 100.0, (0.0) | 41.05, (3.41) | 59.68,  (9.86) |  | 64.48, (11.01) | 3.93, (6.64) | 89.97, (8.51) | 41.68, (6.62) | 56.51,  (15.0) |  | 70.13, (9.66) | 0.0, (0.0) | 100.0, (0.0) | 41.05, (3.41) | 49.34,  (12.26) |
| **PC =3** | 70.13, (9.66) | 0.0, (0.0) | 100.0, (0.0) | 41.05, (3.41) | 62.03,  (11.24) |  | 70.13, (9.66) | 0.0, (0.0) | 100.0, (0.0) | 41.05, (3.41) | 57.88,  (12.62) |  | 64.83, (11.33) | 7.08, (9.63) | 90.07, (11.99) | 44.17, (10.33) | 57.88,  (15.29) |  | 70.13, (9.66) | 0.0, (0.0) | 100.0, (0.0) | 41.05, (3.41) | 50.96,  (20.16) |
| **PC=5** | 70.13, (9.66) | 0.0, (0.0) | 100.0, (0.0) | 41.05, (3.41) | 61.11,  (11.94) |  | 70.13, (9.66) | 0.0, (0.0) | 100.0, (0.0) | 41.05, (3.41) | 57.74,  (9.85) |  | 68.08, (9.33) | 6.25, (11.33) | 95.57, (5.56) | 44.02, (8.39) | 55.69,  (13.47) |  | 70.13, (9.66) | 0.0, (0.0) | 100.0, (0.0) | 41.05, (3.41) | 55.77,  (19.64) |
| **PC=10** | 70.13, (9.66) | 0.0, (0.0) | 100.0, (0.0) | 41.05, (3.41) | 61.73,  (11.44) |  | 70.13, (9.66) | 0.0, (0.0) | 100.0, (0.0) | 41.05, (3.41) | 58.71,  (7.54) |  | **66.45, (7.32)** | **11.25, (20.79)** | **92.55, (7.83)** | **44.76, (8.77)** | **53.46,**  **(9.56)** |  | **69.73, (9.84)** | **1.25, (3.95)** | **98.82, (3.72)** | **41.76, (3.96)** | **49.25,**  **(17.68)** |
| **PC=15** | 70.13, (9.66) | 0.0, (0.0) | 100.0, (0.0) | 41.05, (3.41) | 62.03,  (11.24) |  | **70.13, (9.47)** | **1.25, (3.95)** | **99.47, (1.66)** | **42.13, (4.96)** | **47.12,**  **(10.91)** |  | 65.67, (7.03) | 1.25, (3.95) | 93.52, (5.94) | 40.39, (3.63) | 52.55,  (13.58) |  | 70.13, (9.66) | 0.0, (0.0) | 100.0, (0.0) | 41.05, (3.41) | 55.52,  (10.35) |
| **PC=20** | 70.13, (9.66) | 0.0, (0.0) | 100.0, (0.0) | 41.05, (3.41) | 62.03,  (11.24) |  | 67.70, (9.64) | 2.50, (5.27) | 95.48, (6.47) | 41.92, (4.56) | 58.40,  (11.61) |  | 68.90, (9.22) | 0.0, (0.0) | 98.28, (3.66) | 40.62, (3.47) | 56.65,  (12.13) |  | 70.13, (9.66) | 0.0, (0.0) | 100.0, (0.0) | 41.05, (3.41) | 49.81,  (10.70) |

* The best-performing classifiers for each clinical sign are marked in bold in the above Table.

* LR: logistic regression; RF: random forest; KNN: k nearest neighbor; SVM: support vector machine; Acc: accuracy; SN: sensitivity, SP: specificity, PC: principal component, AUC: area under the curve

**Supplementary Table S12:** Results Obtained Using K-mer Input Representation of DNA Sequences for Abortion (Baseline=52.22)

| **Classifier** | **LR** | | | | | | **RF** | | | | | **KNN** | | | | | | **SVM** | | | | | |
| --- | --- | --- | --- | --- | --- | --- | --- | --- | --- | --- | --- | --- | --- | --- | --- | --- | --- | --- | --- | --- | --- | --- | --- |
| **K-mer** | **Acc** | **SN** | **SP** | **F1-score** | **AUC** |  | **Acc** | **SN** | **SP** | **F1-score** | **AUC** |  | **Acc** | **SN** | **SP** | **F1-score** | **AUC** |  | **Acc** | **SN** | **SP** | **F1-score** | **AUC** |
| **K=2** | 66.78, (11.81) | 60.89, (15.93) | 72.51, (14.50) | 65.82, (11.94) | 70.46,  (12.18) |  | 57.78, (10.15) | 49.72, (20.08) | 65.13, (18.16) | 55.90, (9.74) | 64.48,  (11.10) |  | 55.43, (7.36) | 50.49, (13.19) | 60.80, (16.52) | 54.28, (7.33) | 63.85,  (10.52) |  | 59.88, (10.71) | 57.46, (16.66) | 63.50, (16.30) | 58.91, (10.55) | 69.59,  (12.54) |
| **K=3** | **67.55, (10.48)** | **69.45, (17.31)** | **67.14, (12.20)** | **67.00, (10.55)** | **69.88,**  **(12.09)** |  | 65.92, (7.68) | 70.14, (12.56) | 64.07, (13.37) | 65.60, (7.64) | 69.75,  (9.12) |  | 59.85, (10.06) | 62.53, (13.13) | 57.92, (11.98) | 59.34, (9.78) | 66.99,  (11.89) |  | 63.53, (9.16) | 69.92, (13.98) | 58.31, (12.42) | 62.89, (8.90) | 68.85,  (10.24) |
| **K=4** | 65.10, (12.06) | 72.85, (17.02) | 58.28, (12.48) | 64.48, (11.99) | 69.08,  (14.12) |  | 63.42, (11.10) | 63.70, (23.80) | 65.58, (12.24) | 62.79, (11.64) | 68.46,  (10.40) |  | 62.62, (11.74) | 71.51, (14.51) | 54.92, (13.23) | 61.95, (11.61) | 67.95,  (13.0) |  | 63.43, (10.28) | 72.71, (14.71) | 55.97, (10.43) | 62.96, (10.23) | 70.81,  (11.21) |
| **K=5** | 58.62, (8.30) | 61.46, (14.03) | 56.96, (12.07) | 58.08, (8.58) | 64.90,  (10.67) |  | **69.53, (11.82)** | **76.03, (13.70)** | **65.16, (15.38)** | **69.19, (11.78)** | **71.67,**  **(11.76)** |  | **65.47, (11.89)** | **77.44, (14.55)** | **55.36, (12.63)** | **64.84, (11.68)** | **65.84,**  **(12.58)** |  | **64.23, (10.53)** | **65.99, (12.45)** | **64.38, (14.83)** | **63.89, (10.34)** | **70.63,**  **(8.52)** |
| **K=6** | 63.42, (10.97) | 66.42, (13.45) | 61.07, (11.65) | 63.01, (11.02) | 69.25,  (11.96) |  | 65.88, (10.66) | 71.93, (12.57) | 62.04, (12.86) | 65.64, (10.51) | 71.78,  (8.75) |  | 62.33, (10.6) | 54.52, (17.71) | 70.53, (10.37) | 61.28, (10.92) | 68.49,  (10.80) |  | 62.67, (9.86) | 60.81, (10.73) | 65.42, (11.68) | 62.35, (10.05) | 71.48,  (10.15) |

* The best-performing classifiers for each clinical sign are marked in bold in the above Table.

* LR: logistic regression; RF: random forest; KNN: k nearest neighbor; SVM: support vector machine; Acc: accuracy; SN: sensitivity, SP: specificity, PC: principal component, AUC: area under the curve

**Supplementary Table S13:** Results Obtained Using K-mer Input Representation of DNA Sequences For PWM (Baseline=50.60)

| **Classifier** | **LR** | | | | | | **RF** | | | | | **KNN** | | | | | | **SVM** | | | | | |
| --- | --- | --- | --- | --- | --- | --- | --- | --- | --- | --- | --- | --- | --- | --- | --- | --- | --- | --- | --- | --- | --- | --- | --- |
| **K-mer** | **Acc** | **SN** | **SP** | **F1-score** | **AUC** |  | **Acc** | **SN** | **SP** | **F1-score** | **AUC** |  | **Acc** | **SN** | **SP** | **F1-score** | **AUC** |  | **Acc** | **SN** | **SP** | **F1-score** | **AUC** |
| **K=2** | 56.27, (10.98) | 60.19, (16.76) | 54.29, (11.84) | 55.70, (10.75) | 59.80,  (10.25) |  | 56.28, (8.45) | 67.87, (18.37) | 49.31, (18.82) | 55.11, (9.13) | 60.90,  (6.43) |  | 54.62, (9.60) | 60.68, (13.15) | 50.76, (13.26) | 54.17, (9.40) | 59.23,  (11.21) |  | **63.58, (9.13)** | **80.64, (10.20)** | **46.15, (9.38)** | **61.74, (8.57)** | **62.0,**  **(12.15)** |
| **K=3** | 58.32, (8.60) | 67.73, (18.37) | 49.92, (9.94) | 57.08, (9.04) | 61.55,  (11.40) |  | 58.32, (4.07) | 67.77, (15.38) | 50.61, (16.95) | 56.84, (4.92) | 65.75,  (6.06) |  | 55.05, (11.80) | 60.87, (14.83) | 50.81, (13.63) | 54.49, (11.28) | 58.70,  (12.14) |  | 58.35, (11.15) | 69.53, (22.07) | 49.21, (13.34) | 57.06, (11.58) | 62.38,  (13.16) |
| **K=4** | 58.3, (8.93) | 63.86, (18.60) | 53.82, (5.82) | 57.48, (8.99) | 61.62,  (11.14) |  | 57.07, (8.95) | 65.86, (15.91) | 49.57, (14.25) | 55.90, (8.59) | 61.25,  (8.56) |  | 57.87, (7.84) | 64.13, (12.49) | 52.92, (12.50) | 57.08, (8.19) | 60.75,  (7.77) |  | 57.87, (6.24) | 71.46, (14.10) | 43.86, (12.39) | 55.74, (5.95) | 65.38,  (6.74) |
| **K=5** | **61.10, (8.74)** | **63.31, (10.84)** | **59.54, (13.14)** | **60.35, (8.65)** | **65.76,**  **(9.15)** |  | 58.67, (7.54) | 61.86, (14.82) | 56.11, (13.74) | 57.44, (7.97) | 63.65,  (9.14) |  | 56.67, (5.47) | 75.12, (14.49) | 39.49, (6.41) | 54.74, (5.10) | 59.58,  (8.07) |  | 59.93, (7.30) | 69.20, (8.70) | 52.56, (12.98) | 59.21, (6.89) | 67.36,  (8.25) |
| **K=6** | 58.75, (6.49) | 65.66, (13.10) | 51.52, (11.97) | 57.38, (6.63) | 62.64,  (8.12) |  | **64.35, (7.37)** | **68.89, (17.07)** | **62.39, (11.30)** | **63.80, (7.33)** | **68.26,**  **(8.31)** |  | **59.10, (5.89)** | **71.29, (15.17)** | **47.42, (9.39)** | **57.67, (5.80)** | **61.74,**  **(4.72)** |  | 59.95, (10.61) | 64.93, (14.630) | 55.43, (9.98) | 59.25, (9.94) | 69.35,  (8.24) |

* The best-performing classifiers for each clinical sign are marked in bold in the above Table.

* LR: logistic regression; RF: random forest; KNN: k nearest neighbor; SVM: support vector machine; Acc: accuracy; SN: sensitivity, SP: specificity, AUC: area under the curve

**Supplementary Table S14:** Results Obtained Using K-mer Input Representation of DNA Sequences for Sow Mortality (Baseline=70.04)

| **Classifier** | **LR** | | | | | | **RF** | | | | | **KNN** | | | | | | **SVM** | | | | | |
| --- | --- | --- | --- | --- | --- | --- | --- | --- | --- | --- | --- | --- | --- | --- | --- | --- | --- | --- | --- | --- | --- | --- | --- |
| **K-mer** | **Acc** | **SN** | **SP** | **F1-score** | **AUC** |  | **Acc** | **SN** | **SP** | **F1-score** | **AUC** |  | **Acc** | **SN** | **SP** | **F1-score** | **AUC** |  | **Acc** | **SN** | **SP** | **F1-score** | **AUC** |
| **K=2** | 70.13, (9.66) | 0.0, (0.0) | 100.0, (0.0) | 41.05, (3.41) | 51.83,  (8.89) |  | 68.12, (7.76) | 16.94, (12.25) | 91.31, (8.93) | 50.48, (8.35) | 65.86,  (11.72) |  | 65.65, (9.67) | 24.77, (23.02) | 85.30, (13.20) | 50.48, (10.98) | 60.0,  (10.90) |  | 70.13, (9.66) | 0.0, (0.0) | 100.0, (0.0) | 41.05, (3.41) | 49.97,  (19.38) |
| **K=3** | 69.73, (9.47) | 0.0, (0.0) | 99.47, (1.66) | 40.92, (3.34) | 51.90,  (6.06) |  | 69.23, (7.81) | 22.33, (17.06) | 89.77, (5.10) | 53.56, (11.34) | 63.61,  (13.86) |  | 62.35, (9.77) | 28.27, (25.74) | 80.18, (11.05) | 50.06, (14.15) | 60.26,  (12.54) |  | 70.13, (9.66) | 0.0, (0.0) | 100.0, (0.0) | 41.05, (3.41) | 45.66,  (12.50) |
| **K=4** | **67.68, (9.72)** | **2.92, (6.23)** | **95.52, (5.82)** | **42.26, (6.10)** | **53.50,**  **(10.29)** |  | 68.85, (8.19) | 19.67, (22.70) | 91.82, (7.73) | 51.12, (11.78) | 65.90,  (9.58) |  | 61.88, (9.56) | 18.08, (19.46) | 83.54, (14.12) | 46.31, (8.56) | 58.91,  (10.03) |  | 68.85, (7.97) | 5.83, (11.15) | 96.90, (6.55) | 44.15, (7.67) | 55.07,  (8.71) |
| **K=5** | 66.02, (7.92) | 0.0, (0.0) | 94.74, (8.98) | 39.64, (2.95) | 55.20,  (9.61) |  | **68.85, (10.26)** | **27.94, (20.7)** | **88.64, (7.13)** | **55.20, (12.15)** | **67.60,**  **(9.59)** |  | 61.57, (7.47) | 33.83, (17.26) | 75.41, (15.22) | 51.77, (5.75) | 59.99,  (11.32) |  | 67.28, (10.07) | 13.75, (19.45) | 90.83, (8.62) | 47.30, (12.06) | 60.08,  (6.85) |
| **K=6** | 65.68, (13.12) | 4.58, (10.84) | 91.45, (11.81) | 41.65, (7.43) | 57.62,  (11.25) |  | 66.83, (8.89) | 15.94, (17.54) | 90.10, (8.56) | 48.70, (11.41) | 64.53,  (11.38) |  | **62.40, (8.21)** | **49.81, (25.18)** | **68.41, (11.52)** | **55.96, (10.93)** | **63.49,**  **(11.24)** |  | **69.70, (8.32)** | **17.67, (20.13)** | **93.24, (7.40)** | **50.35, (11.09)** | **66.02,**  **(9.66)** |

* The best-performing classifiers for each clinical sign are marked in bold in the above Table.

* LR: logistic regression; RF: random forest; KNN: k nearest neighbor; SVM: support vector machine; Acc: accuracy; SN: sensitivity, SP: specificity, AUC: area under the curve

**Supplementary Table S15:** Results Obtained Using K-mer Input Representation of Amino Acid Sequences for Abortion (Baseline=52.22)

| **Classifier** | **LR** | | | | | | **RF** | | | | | **KNN** | | | | | | **SVM** | | | | | |
| --- | --- | --- | --- | --- | --- | --- | --- | --- | --- | --- | --- | --- | --- | --- | --- | --- | --- | --- | --- | --- | --- | --- | --- |
| **K-mer** | **Acc** | **SN** | **SP** | **F1-score** | **AUC** |  | **Acc** | **SN** | **SP** | **F1-score** | **AUC** |  | **Acc** | **SN** | **SP** | **F1-score** | **AUC** |  | **Acc** | **SN** | **SP** | **F1-score** | **AUC** |
| **K=2** | 63.45, (10.92) | 72.78, (16.86) | 56.23, (12.04) | 62.94, (10.82) | 69.09, (9.04) |  | 64.63, (10.42) | 74.02, (18.39) | 56.54, (13.17) | 63.65, (10.55) | 67.75, (13.09) |  | 62.23, (11.57) | 75.88, (12.70) | 50.18, (13.15) | 61.29, (11.28) | 68.51, (7.90) |  | 62.67, (10.11) | 69.24, (14.64) | 56.63, (12.80) | 61.89, (9.61) | 68.48, (5.67) |
| **K=3** | **67.12, (10.25)** | **72.92, (16.42)** | **63.42, (11.44)** | **66.73, (10.19)** | **69.70, (9.14)** |  | 64.65, (10.50) | 71.33, (14.30) | 59.16, (14.35) | 63.96, (10.32) | 70.45, (10.68) |  | 61.87, (11.09) | 61.94, (14.70) | 63.34, (13.23) | 61.45, (10.91) | 69.39, (11.99) |  | **67.15, (5.87)** | **66.10, (13.70)** | **69.56, (11.20)** | **66.60, (6.01)** | **69.93, (7.09)** |
| **K=4** | 61.43, (9.32) | 65.62, (12.70) | 57.97, (10.55) | 60.91, (9.22) | 68.71, (10.17) |  | 64.67, (10.68) | 66.36, (18.96) | 64.73, (16.19) | 63.94, (10.69) | 70.96, (13.63) |  | 61.42, (9.98) | 56.62, (23.06) | 69.05, (19.36) | 60.13, (10.17) | 68.65, (8.78) |  | 66.30,  (9.79) | 69.04, (12.88) | 65.19, (13.79) | 65.90, (9.93) | 71.29, (7.86) |
| **K=5** | 59.45, (8.88) | 60.45, (11.62) | 59.55, (10.90) | 59.09, (8.93) | 69.23, (10.67) |  | 65.88, (9.22) | 74.78, (14.19) | 57.73, (11.05) | 65.06, (9.28) | 70.99, (8.78) |  | 61.05, (9.74) | 58.27, (18.69) | 64.95, (12.61) | 60.31, (10.01) | 67.70, (12.41) |  | 64.28, (10.72) | 64.96, (15.56) | 63.37, (12.83) | 63.41, (11.15) | 67.98, (11.30) |
| **K=6** | 63.83, (11.02) | 69.32, (11.69) | 60.56, (13.31) | 63.59, (10.99) | 69.60, (10.54) |  | **66.28, (8.93)** | **68.51, (15.05)** | **66.13, (13.14)** | **65.91, (8.90)** | **71.48, (10.44)** |  | **63.40, (13.71)** | **58.11, (17.59)** | **68.84, (18.53)** | **62.40, (13.48)** | **69.50, (14.19)** |  | 65.47, (9.76) | 67.05, (9.15) | 64.61, (12.41) | 65.09, (9.57) | 67.39, (12.97) |

* The best-performing classifiers for each clinical sign are marked in bold in the above Table.

* LR: logistic regression; RF: random forest; KNN: k nearest neighbor; SVM: support vector machine; Acc: accuracy; SN: sensitivity, SP: specificity, AUC: area under the curve

**Supplementary Table S16:** Results Obtained Using K-mer Input Representation of Amino Acid Sequences For PWM (Baseline=50.60)

| **Classifier** | **LR** | | | | | | **RF** | | | | | **KNN** | | | | | | **SVM** | | | | | |
| --- | --- | --- | --- | --- | --- | --- | --- | --- | --- | --- | --- | --- | --- | --- | --- | --- | --- | --- | --- | --- | --- | --- | --- |
| **K-mer** | **Acc** | **SN** | **SP** | **F1-score** | **AUC** |  | **Acc** | **SN** | **SP** | **F1-score** | **AUC** |  | **Acc** | **SN** | **SP** | **F1-score** | **AUC** |  | **Acc** | **SN** | **SP** | **F1-score** | **AUC** |
| **K=2** | 58.68, (7.97) | 64.45, (18.24) | 54.37, (15.73) | 57.59, (8.55) | 63.31, (10.42) |  | 60.27, (5.77) | 70.13, (11.22) | 50.68, (9.02) | 59.08, (5.12) | 66.76, (6.97) |  | 56.70, (8.37) | 67.95, (6.08) | 46.99, (14.29) | 55.66, (8.30) | 59.89, (6.27) |  | 55.78, (10.38) | 59.22, (14.80) | 50.14, (12.30) | 54.33, (9.82) | 60.58, (9.56) |
| **K=3** | 66.85, (7.80) | 71.97, (13.84) | 62.12, (6.42) | 66.10, (7.33) | 73.91, (4.64) |  | 62.75, (4.13) | 74.10, (10.27) | 52.08, (12.45) | 61.40, (3.68) | 68.84, (5.54) |  | 53.03, (5.36) | 56.96, (25.3) | 56.31, (20.86) | 52.35, (5.54) | 60.25, (12.24) |  | 65.15, (5.62) | 71.18, (9.89) | 59.47, (5.73) | 64.37, (4.95) | 69.93, (2.99) |
| **K=4** | 66.37, (8.23) | 74.48, (14.75) | 60.45, (11.61) | 65.76, (7.58) | 72.05, (4.47) |  | **64.72, (5.79)** | **73.76, (14.18)** | **58.00, (10.26)** | **64.03, (4.94)** | **69.75, (5.03)** |  | **63.27, (9.81)** | **70.53, (16.75)** | **59.85, (19.81)** | **62.58, (10.18)** | **67.17, (11.19)** |  | **68.38, (5.56)** | **68.88, (13.57)** | **68.01, (9.63)** | **67.47, (5.08)** | **70.88, (5.08)** |
| **K=5** | **69.25, (6.80)** | **76.07, (11.84)** | **62.12, (6.09)** | **68.33, (6.45)** | **73.18, (4.30)** |  | 64.80, (8.86) | 75.18, (13.89) | 55.90, (13.01) | 63.85, (8.54) | 69.84, (7.05) |  | 58.77, (10.04) | 68.79, (18.59) | 52.99, (15.75) | 58.18, (9.74) | 63.46, (9.98) |  | 64.75, (7.69) | 72.68, (14.46) | 56.89, (6.80) | 63.71, (7.11) | 70.68, (4.99) |
| **K=6** | 66.80, (5.27) | 73.59, (10.94) | 60.43, (10.88) | 65.82, (4.88) | 70.91, (2.59) |  | 63.53, (6.69) | 73.51, (15.28) | 55.35, (9.53) | 62.70, (5.74) | 68.46, (5.16) |  | 62.80, (6.81) | 74.07, (17.59) | 52.73, (11.76) | 61.50, (6.42) | 64.12, (6.26) |  | 64.35, (11.15) | 73.17, (15.33) | 55.91, (10.01) | 63.45, (10.78) | 68.31, (12.07) |

* The best-performing classifiers for each clinical sign are marked in bold in the above Table.

* LR: logistic regression; RF: random forest; KNN: k nearest neighbor; SVM: support vector machine; Acc: accuracy; SN: sensitivity, SP: specificity, AUC: area under the curve

**Supplementary Table S17:** Results Obtained Using K-mer Input Representation of Amino Acid Sequences for Sow Mortality (Baseline=70.04)

| **Classifier** | **LR** | | | | | | **RF** | | | | | **KNN** | | | | | | **SVM** | | | | | |
| --- | --- | --- | --- | --- | --- | --- | --- | --- | --- | --- | --- | --- | --- | --- | --- | --- | --- | --- | --- | --- | --- | --- | --- |
| **K-mer** | **Acc** | **SN** | **SP** | **F1-score** | **AUC** |  | **Acc** | **SN** | **SP** | **F1-score** | **AUC** |  | **Acc** | **SN** | **SP** | **F1-score** | **AUC** |  | **Acc** | **SN** | **SP** | **F1-score** | **AUC** |
| **K=2** | 69.20, (9.25) | 7.82, (13.14) | 95.52, (5.34) | 45.94, (9.72) | 50.37, (17.90) |  | 69.60, (10.86) | 0.0, (0.0) | 98.57, (4.52) | 40.81, (4.01) | 54.38, (14.15) |  | 69.20, (9.62) | 0.0, (0.0) | 98.23, (2.90) | 40.72, (3.49) | 48.42, (14.73) |  | 70.40, (9.28) | 0.0, (0.0) | 100.0, (0.0) | 41.15, (3.30) | 40.02, (11.84) |
| **K=3** | 68.43, (9.01) | 22.25, (21.94) | 89.81, (6.96) | 52.22, (11.42) | 65.33, (9.99) |  | 70.08, (9.34) | 22.42, (21.27) | 92.04, (6.26) | 53.59, (11.51) | 66.01, (9.96) |  | **68.88, (9.95)** | **10.83, (15.61)** | **94.50, (7.32)** | **47.07, (10.25)** | **59.75, (12.46)** |  | 71.37, (12.21) | 11.83, (20.37) | 98.12, (3.09) | 49.76, (15.70) | 70.58, (5.49) |
| **K=4** | **68.02, (9.58)** | **22.83, (21.37)** | **89.47, (6.91)** | **52.59, (12.33)** | **65.35, (5.96)** |  | 68.03, (10.0) | 21.17, (21.47) | 89.69, (6.97) | 51.81, (12.28) | 65.96, (6.67) |  | 69.32, (9.11) | 6.17, (8.23) | 95.96, (3.95) | 45.23, (6.50) | 58.73, (12.93) |  | 72.18, (12.20) | 14.50, (20.03) | 98.12, (3.09) | 52.16, (15.70) | 69.32, (5.90) |
| **K=5** | 67.58, (9.71) | 21.58, (19.92) | 89.62, (9.48) | 52.06, (11.44) | 64.01, (12.29) |  | 66.40, (10.49) | 18.67, (20.24) | 88.78, (11.28) | 49.41, (10.65) | 63.86, (8.06) |  | 68.87, (7.65) | 8.25, (11.85) | 95.76, (5.21) | 45.80, (8.41) | 62.33, (11.61) |  | 71.35, (11.96) | 16.17, (20.90) | 96.45, (5.51) | 52.21, (15.73) | 70.77, (7.25) |
| **K=6** | 68.03, (10.79) | 20.92, (21.28) | 89.70, (6.31) | 51.86, (13.30) | 67.90, (8.15) |  | **71.72, (11.40)** | **22.58, (22.18)** | **94.29, (5.97)** | **55.18, (15.04)** | **67.60, (10.04)** |  | 68.50, (8.13) | 3.33, (10.54) | 97.46, (4.73) | 41.92, (6.31) | 60.23, (8.53) |  | **72.17, (11.79)** | **17.42, (20.19)** | **97.01, (4.15)** | **53.65, (15.25)** | **71.35, (7.66)** |

* The best-performing classifiers for each clinical sign are marked in bold in the above Table.

* LR: logistic regression; RF: random forest; KNN: k nearest neighbor; SVM: support vector machine; Acc: accuracy; SN: sensitivity, SP: specificity, AUC: area under the curve
